# Cortactin is in a complex with VE-cadherin and is required for endothelial adherens junction stability through Rap1/Rac1 activation

**DOI:** 10.1038/s41598-024-51269-3

**Published:** 2024-01-12

**Authors:** Sina Moztarzadeh, Sara Sepic, Ibrahim Hamad, Jens Waschke, Mariya Y. Radeva, Alexander García-Ponce

**Affiliations:** Chair of Vegetative Anatomy, Faculty of Medicine, Ludwig-Maximilians-University (LMU) Munich, Pettenkoferstraße 11, 80336 Munich, Germany

**Keywords:** Cell biology, Cell adhesion, Adherens junctions, Cardiovascular biology

## Abstract

Vascular permeability is mediated by Cortactin (Cttn) and regulated by several molecules including cyclic-adenosine-monophosphate, small Rho family GTPases and the actin cytoskeleton. However, it is unclear whether Cttn directly interacts with any of the junctional components or if Cttn intervenes with signaling pathways affecting the intercellular contacts and the cytoskeleton. To address these questions, we employed immortalized microvascular myocardial endothelial cells derived from wild-type and Cttn-knock-out mice. We found that lack of Cttn compromised barrier integrity due to fragmented membrane distribution of different junctional proteins. Moreover, immunoprecipitations revealed that Cttn is within the VE-cadherin-based adherens junction complex. In addition, lack of Cttn slowed-down barrier recovery after Ca^2+^ repletion. The role of Cttn for cAMP-mediated endothelial barrier regulation was analyzed using Forskolin/Rolipram. In contrast to Cttn-KO, WT cells reacted with increased transendothelial electrical resistance. Absence of Cttn disturbed Rap1 and Rac1 activation in Cttn-depleted cells. Surprisingly, despite the absence of Cttn, direct activation of Rac1/Cdc42/RhoA by CN04 increased barrier resistance and induced well-defined cortical actin and intracellular actin bundles. In summary, our data show that Cttn is required for basal barrier integrity by allowing proper membrane distribution of junctional proteins and for cAMP–mediated activation of the Rap1/Rac1 signaling pathway.

## Introduction

The endothelium functions as gate-keeper between the blood vessels and the underlying tissue, thereby controlling the transport of nutrients, cells and proteins circulating in the blood^1,2^. This process involves the opening and closure of cell–cell contacts^3^, where several molecules such as the second messenger, cyclic adenosine monophosphate (cAMP), Ras homologous small guanosine-triphosphatases (Rho-GTPases) and endothelial junctional molecules are involved^4,5^. Endothelial junctions, formed by transmembrane adhesive proteins consist of adherens junctions (AJs), formed by homophilic interaction between vascular endothelial cadherin (VE-cadherin); and tight junctions (TJ), predominantly comprised of claudins, like the endothelial-specific Claudin-5. These proteins, not only mediate the interactions between neighboring cells, but are also linked through their cytoplasmic domains to the actin cytoskeleton via adaptor proteins like the catenin family members (α-catenin, β-catenin and γ –catenin, also known as plakoglobin) and Zonula Occludens-1 (ZO-1)^6,7^. Actin is the main component of the cytoskeleton, actin dynamics have been demonstrated to be critical for endothelial barrier function in vivo and for cadherin-mediated binding and AJ stability in vitro^8^. Polymerization and interlinking of actin filaments provide critical functions to the cell including mobility, cell adhesion and mechanical support to preserve the cell shape^9,10^. The ever-changing dynamics of this network are governed not only by different signaling molecules but also by actin-binding proteins (ABPs) such as Cortactin and Adducin^9,11–13^. Under pathophysiological conditions such as the exacerbated inflammatory response seen in sepsis, enhanced actomyosin contractility associates with increased vascular permeability and death^14–17^. Therefore, besides intercellular junctional integrity, dynamics of the actin cytoskeleton and ABPs are critical for endothelial barrier stability.

Cttn is a multifunctional protein; it participates in lamellipodia, invadopodia, cell–cell adhesion and cell migration. The structure of this protein consists of different domains such as a N-terminal, a 6.5 tandem repeat known as the cortactin repeat, an α-Helix, Prolin-rich and Src-homology 3 (SH3)^18,19^ This molecule acts as a platform where different structural and signaling proteins interact. For instance, its N-terminal domain is responsible for actin filament branching by supporting the activation of the Arp2/3 complex^20–22^. Cttn directly binds to F-actin via the tandem repeat, thereby participating in several cellular processes requiring actin. Furthermore, its SH3 domain can interact with E-cadherin in epithelial cells and ZO-1 in mouse tissues^23,24^. These features highlight Cttn as a relevant player in endothelial barrier homeostasis. Previously, it was reported that one of the mechanisms Cttn employs to preserve the endothelial barrier homeostasis involves preventing actin stress fibers polymerization and actomyosin contractility^25,26^. In addition, Cttn supports the efficient secretion of the peptide hormone adrenomedullin (ADM) in the circulation, known to stimulate cAMP production^26–30^. cAMP is another important player in barrier regulation and its enhanced intracellular level has been extensively associated with improved barrier stability^31–34^. This enhancement results in the activation of the small GTPase Rac1, facilitating the translocation of Cttn to the cell periphery, on the one hand, and reducing stress fibers formation on the other^35,36^. Therefore, cAMP has emerged as a master regulator of cadherin-mediated adhesion both in endothelial AJ as well as in desmosomal contacts of the myocardium and of keratinocytes^37^. Moreover, a report from Schnoor et al. associated the lack of Cttn with impaired cAMP-mediated signaling demonstrated by reduced active levels of Rap1^38^. However, the activation state of Rac1 or RhoA and the composition of the intercellular contacts were not investigated in detail in the aforementioned studies.

In the current study, we aimed to further understand the molecular machinery by which Cttn promotes endothelial barrier function and its contribution towards cAMP-mediated signaling. We demonstrated that Cttn facilitates the proper membrane localization of junctional proteins and efficient endothelial barrier recovery, partly involving the regulation of Rap1 and Rac1 via cAMP.

## Results

### The absence of Cttn leads to fragmented intercellular junctions, altered F-actin distribution and impaired barrier function

**Figure 1:**
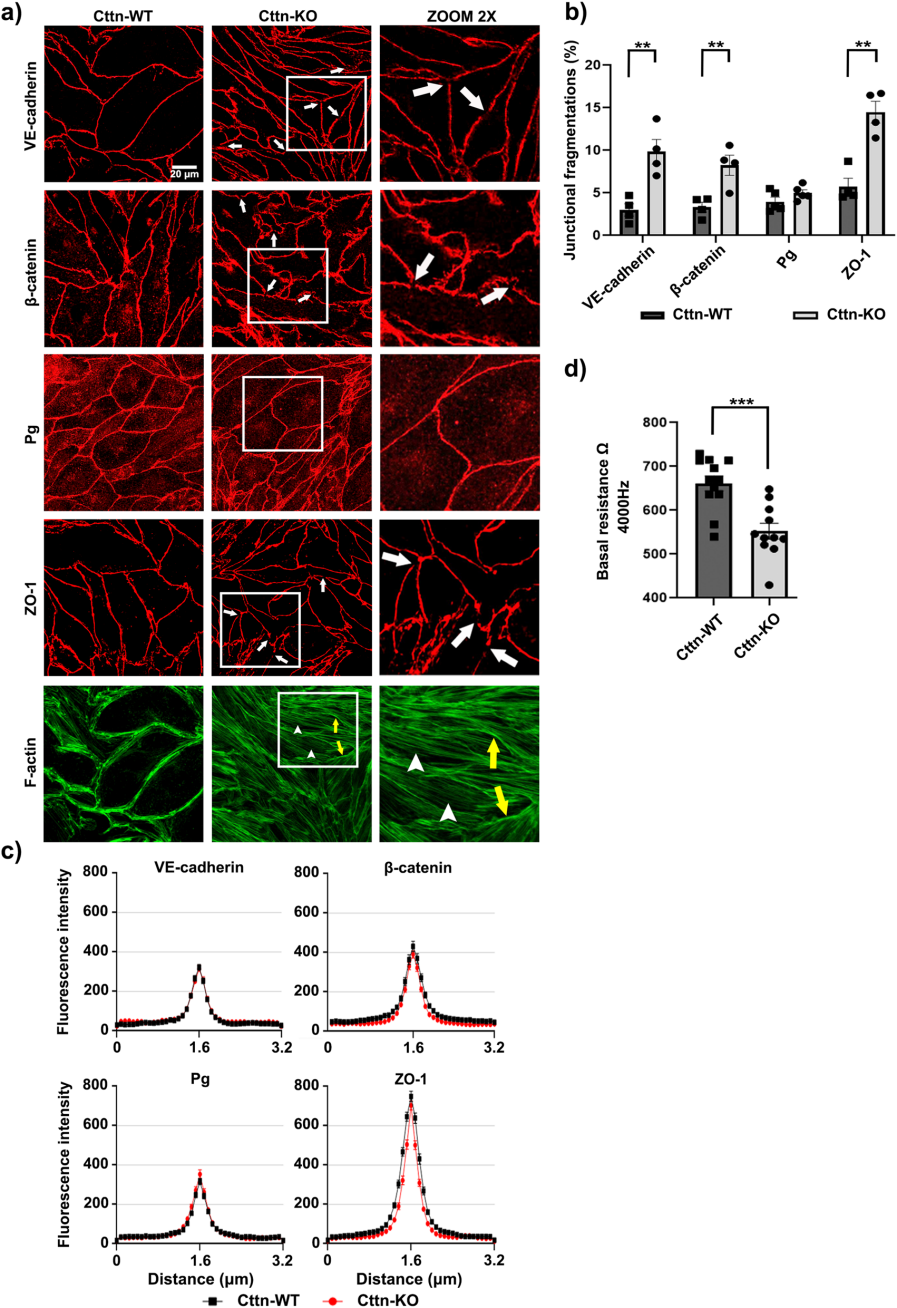
Cttn loss led to junctional fragmentation and compromised barrier integrity. (**a**) Staining of VE-cadherin, β-catenin, Pg, ZO-1 and F-actin. White arrows indicate spots of junctional fragmentation. Yellow arrows show formed cortical actin and arrowheads illustrate intracellular actin fibers. White frames indicate zoomed areas within the Cttn-KO monolayer, N = 4–5. (**b**) Bar graphs depict the quantification of junctional fragmentation for each protein shown in A, N = 4–5. (**c**) Bell-shaped curves depict the pixel intensity of each corresponding junctional protein presented in (**a**), N = 4–5. (**d**) TEER measurements of confluent WT and Cttn-KO cells. The bar diagram represents the values recorded under basal conditions, N = 3. Data are represented as mean ± SEM; **p < 0.01; ***p < 0.001.

**Figure 2:**
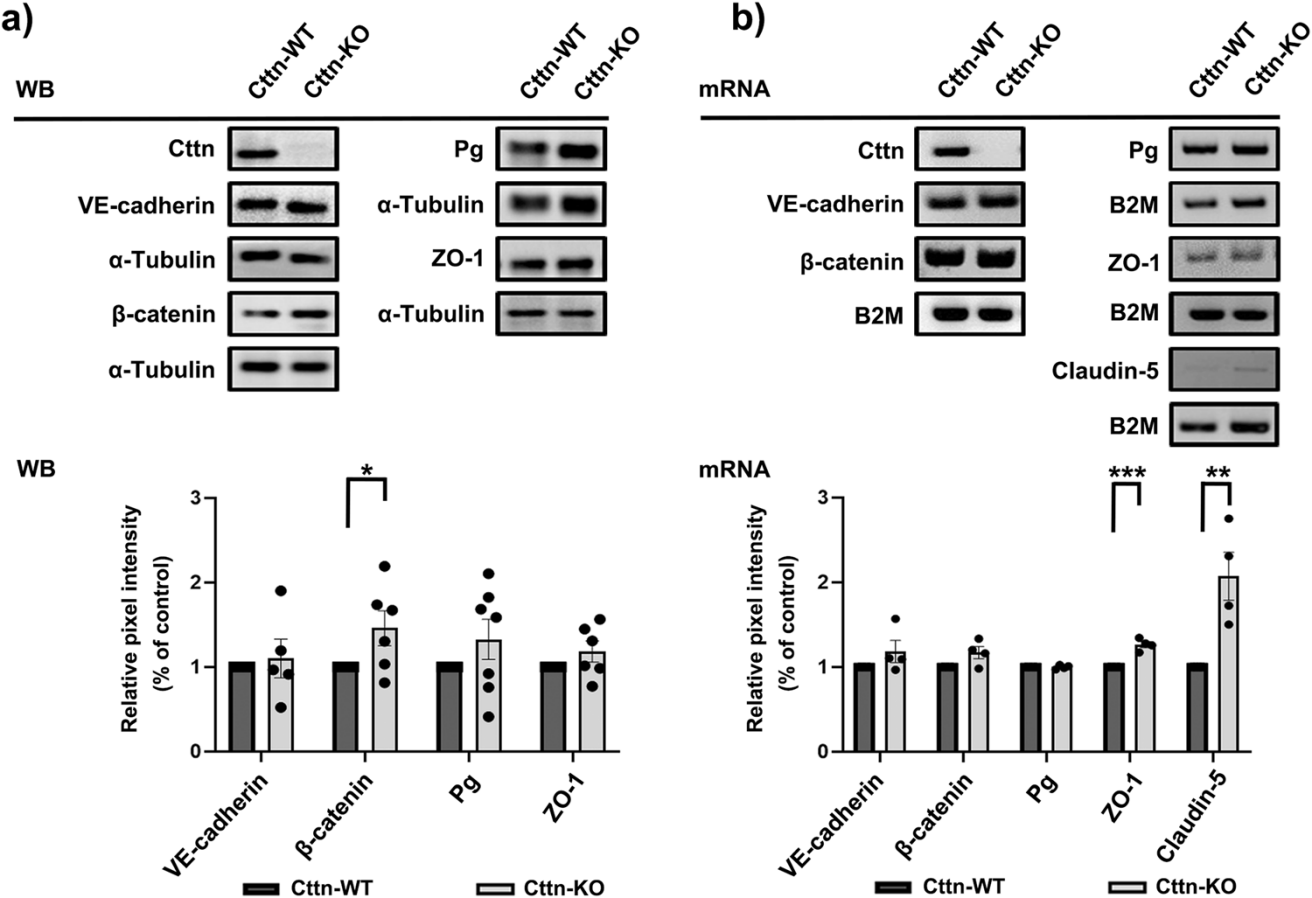
Effect of Cttn deficiency on junctional protein and mRNA levels. (**a**) Western blot analysis of AJ and TJ components. α-tubulin was used as loading control, N = 5–7. (**b**) mRNA levels for Cttn and junctional molecules were analyzed by PCR. β2M house-keeping gene was used as loading control. Bars represent the quantification of the band pixel density normalized to the respective WT control, N = 4. Data are represented as mean ± SEM; *p < 0.05; **p < 0.01.

Although a link between Cttn and endothelial barrier regulation has been previously established^26,38^, it is unclear if Cttn participates in the junctional distribution of endothelial cell contact components. For this reason, we analyzed by immunostainings the membrane distribution of critical TJ and AJ proteins in confluent WT and Cttn-KO Myocardial Endothelial (MyEnd) monolayers (for confirmation of the endothelial phenotype and Cttn protein and gene ablation please refer to Supplementary Fig. 1a, b). In contrast to the previous studies where changes in cell contacts were not determined^26,38^, we noticed that VE-cadherin, β-catenin and ZO-1 showed fragmented membrane distribution in cells lacking Cttn (Fig. 1a, arrows, squares for zoomed areas). No important differences were observed for plakoglobin (Pg) between WT and Cttn-KO cells. Besides, while WT cells had a well-defined and thick cortical actin belt, cells missing Cttn rather exhibited a thin cortical actin and a marked presence of intracellular actin fibers (Fig. 1a, yellow arrows and arrowheads, respectively and zoomed area; information for antibodies is found on Tables 1 and 2). Furthermore, the radically disturbed junctional proteins distribution observed in Cttn-KO cells was verified by calculating the ratio between continuous and fragmented junctions (Fig. 1b). Despite the increased presence of fragments in cells without Cttn, the fluorescence signal intensity and thickness of these molecules were comparable between both cell lines (Fig. 1c). Moreover, the increased junctional fragmentation observed in cells without Cttn appeared to be associated with compromised barrier stability demonstrated by lower electrical resistance (Fig. 1d). These results were further confirmed by calculating the Rb and alpha (α) values from confluent monolayers. Rb is a measure for the electrical resistancein the intercellular clefts directly affected by the tightness of cell–cell contacts. α is a measure for the constraint of current flow within the subcellular cleft^39^. Cttn-KO cells exhibited significantly lower Rb (Supplementary Fig. 2b). On the other hand, α was comparable between both cell lines (Supplementary Fig. 2b). On the other hand, no critical changes in the protein levels of VE-cadherin, Pg and ZO-1 were detected. Interestingly, only the expression of β-catenin was considerably elevated due to the absence of Cttn, probably as part of a compensatory response aiming to preserve endothelial barrier function (Fig. 2a; information for antibodies is found on Tables 1 and 2). The mRNA expression analysis of AJ components was comparable between both cell types, but only Cttn-KO cells displayed important increased levels for ZO-1 and claudin-5 (Fig. 2b; information for primers is found on Table 3).

**Table 1:** List of primary antibodies.

Antibody	Species	Cat. number	Dilution rate/purpose	Company
VE-cadherin	Rabbit	33168	1:1000-WB 1:100-IF	Abcam
α-Tubulin	Mouse	7291	1:1000-WB	Abcam
ZO-1	Rabbit	617300	1:1000-WB 1:100- IF	Thermo Fisher Scientific
β-catenin	Mouse	61054	1:1000-WB 1:100- IF	BD Transduction Laboratories
Plakoglobin	Mouse	61005	1:1000-WB 1:50- IF	Progen
vWF	Rabbit	A0082	1:100- IF	Dako
PECAM-1	Mouse	37676	1:100- IF	Santa Cruz
Rac1	Mouse	BK128	1:1000-WB	Cytoskeleton
RhoA	Rabbit	10749-1-AP	1:1000-WB	Proteintech
Rap1	Rabbit	07916	1:1000-WB	Millipore
Cortactin	Mouse	868102	1:1000-WB 1:100-IF	BIOZOL
IgG control antibody	Rabbit	2729S	1:1000 IP	Cell Signaling Tech

**Table 2:** List of secondary antibodies.

Antibody	Species	Cat number	Dilution Rate/Purpose	Company
Cy3-AffiniPure	Goat Anti-Rabbit IgG (H + L)	111-165-003	1:100-IF	Dianova
Cy3-AffiniPure	Goat Anti-Mouse IgG	115-165-164	1:100-IF	Dianova
Peroxidase-AffiniPure	Goat Anti-Rabbit IgG (H + L)	11-035-003	1:10000-WB	Dianova
Peroxidase-AffiniPure	Goat Anti-Mouse IgG + IgM (H + L)	115-035-068	1:10000-WB	Dianova

**Table 3:** Primers used for RT-PCR.

Target analyzed	5′- > 3′	Amplicon size (bp)
Cttn	FW: GGAAGACTGAGAAGCATGCCT REV: CTGGGATTCGTGCTTCTCTGTC	248
VE-cadherin	FW: GAGTTCACCTTC TGTGAGGAGATG REV: CTTCTGCACCTGCGTGTACAC	329
β-catenin	FW: GAGGACCTACACTTATGAGAAGC REV: GGCAGTCCATAATGAAGGCG	492
Pg	FW: GTTCGGTTACTGAGTTGCTGCCTTGG REV: GGTATTCCAGGTCACCTTGGTTCTG	369
ZO-1	FW: CCACCTCTGTCC AGC TCTTC REV: CACCGG AGTGATGGTTTTCT	248
Claudin-5	FW: GATGTCGTGCGTGGTGCAGAG TAC REV: CTTGTCGTAATCGCCATTGGCCGTG	489
B2M	FW: CAAGTATACTCACGCCACCCAC REV: CATCATGATGCTTGATCACATGTCTC	292

### Cttn is found within the VE-cadherin-based complex and is required for efficient barrier recovery after calcium switch

**Figure 3:**
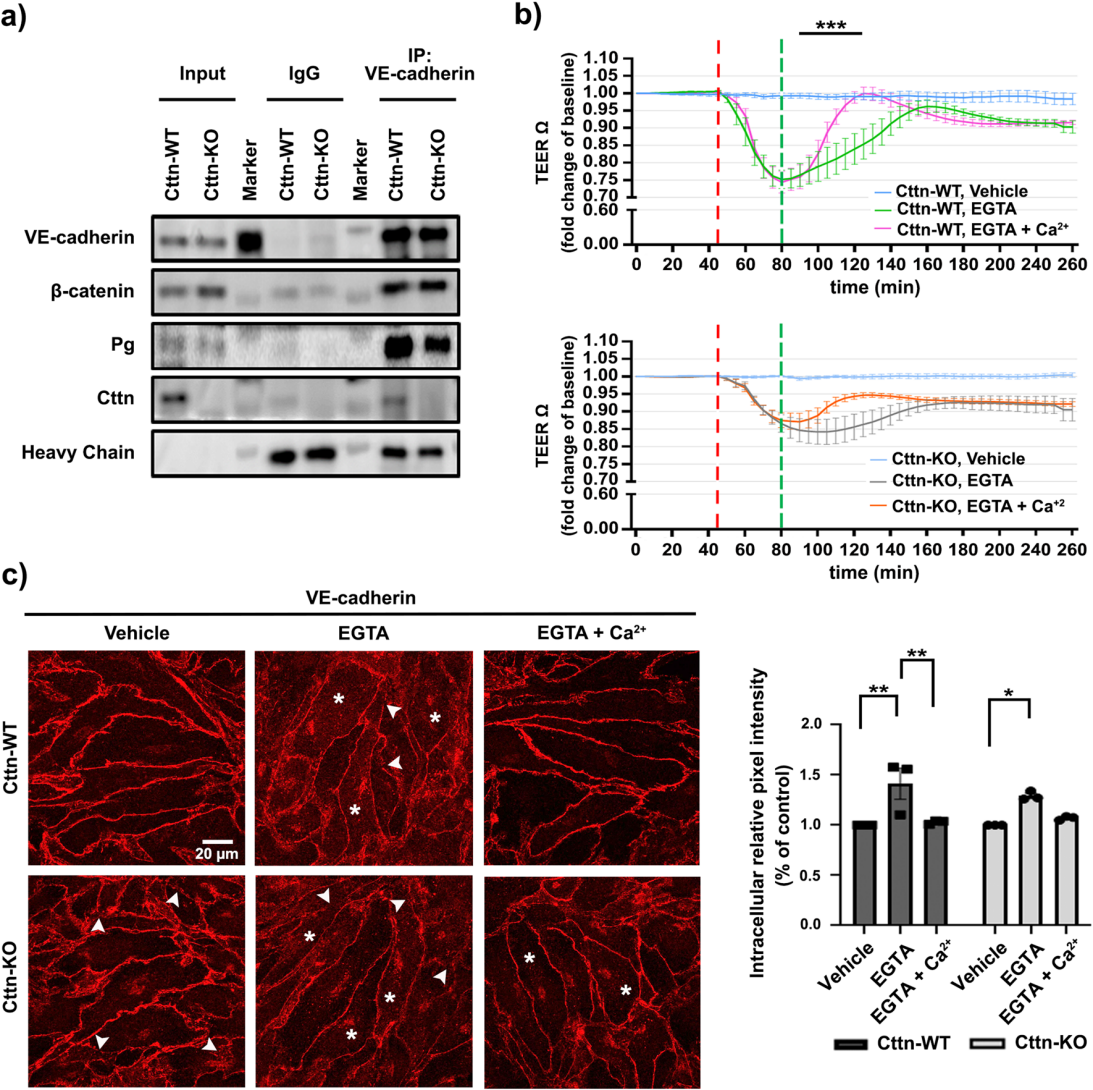
VE-cadherin immunoprecipitation and Ca^+2^ depletion and repletion effect on WT and Cttn-KO cells barrier recovery. (**a**) Representative Western Blots from VE-cadherin immunoprecipitation assays, N = 6. (**b**) TEER analysis, the diagrams represent the data normalized to the corresponding starting point. The segmented lines indicate treatment application, (***) depicts a substantial difference between EGTA and EGTA + Ca ^2+^ in WT cells, N = 4. (**c**) VE-cadherin distribution after depletion and repletion of Ca ^2+^ analyzed by immunofluorescence. Arrowheads highlight spots where the membrane is fragmented, (*) shows intracellular VE-cadherin staining and the diagram represents intracellular intensity quantification. The data are normalized to the respective control, N = 3. Data are represented as mean ± SEM; *p < 0.05; **p < 0.01; ***p < 0.001.

Considering that Cttn is known to interact with ZO-1^24^ and E-cadherin^23^ via the SH3 domain in other cell types, we speculated that it might interact with junctional molecules and be required for the establishment of endothelial cell contacts. To investigate this idea, we performed VE-cadherin immunoprecipitation using confluent cell monolayers. Surprisingly, we found that Cttn and VE-cadherin can be found within the same molecular complex along with β-catenin and Pg (Fig. 3a). To further analyze if Cttn is associated with the stability of the Ca^2+^-dependent VE-cadherin homophilic interaction and endothelial barrier recovery, calcium switch assay combined with TEER was performed. EGTA was used as chelator to remove the extracellular Ca^2+^, thus disrupting pre-stablished AJs. Subsequent addition of Ca^2+^ facilitates junctional re-assembly. Both cell lines reacted to EGTA with a drop in resistance (Fig. 3b, green line for WT; gray line for KO). However, only WT cells were able to quickly recover back to baseline after Ca^2+^ addition (Fig. 3b, purple line). Cttn-KO cells also showed a slight but insignificant recovery after Ca^2+^ (Fig. 3b, orange line). In addition, VE-cadherin dynamics were analyzed after calcium switch. As expected, in vehicle-treated Cttn-KO, a clear fragmented VE-cadherin membrane staining was recognized (Fig. 3c, arrowheads). In both cell lines, treatment with EGTA resulted in diffused intracellular staining of VE-cadherin (Fig. 3c, asterisks “*”) and decreased signal intensity along the junctions. The effect was associated with prominent segmented junctions (Fig. 3c, arrowheads). Following Ca^2+^ repletion, WT cells showed better VE-cadherin membrane distribution and considerably less intracellular signal (Fig. 3c, arrows and bar chart). Cells without Cttn also reacted favorably to repletion of Ca^2+^ by improving VE-cadherin membrane staining, although the intracellular signal was still visible (Fig. 3c, asterisks “*”). Altogether, the data show that the proper membrane translocation of VE-cadherin is partly dependent on its interaction with Cttn.

### Cells lacking Cttn are unable to improve barrier function upon intracellular cAMP increase

**Figure 4:**
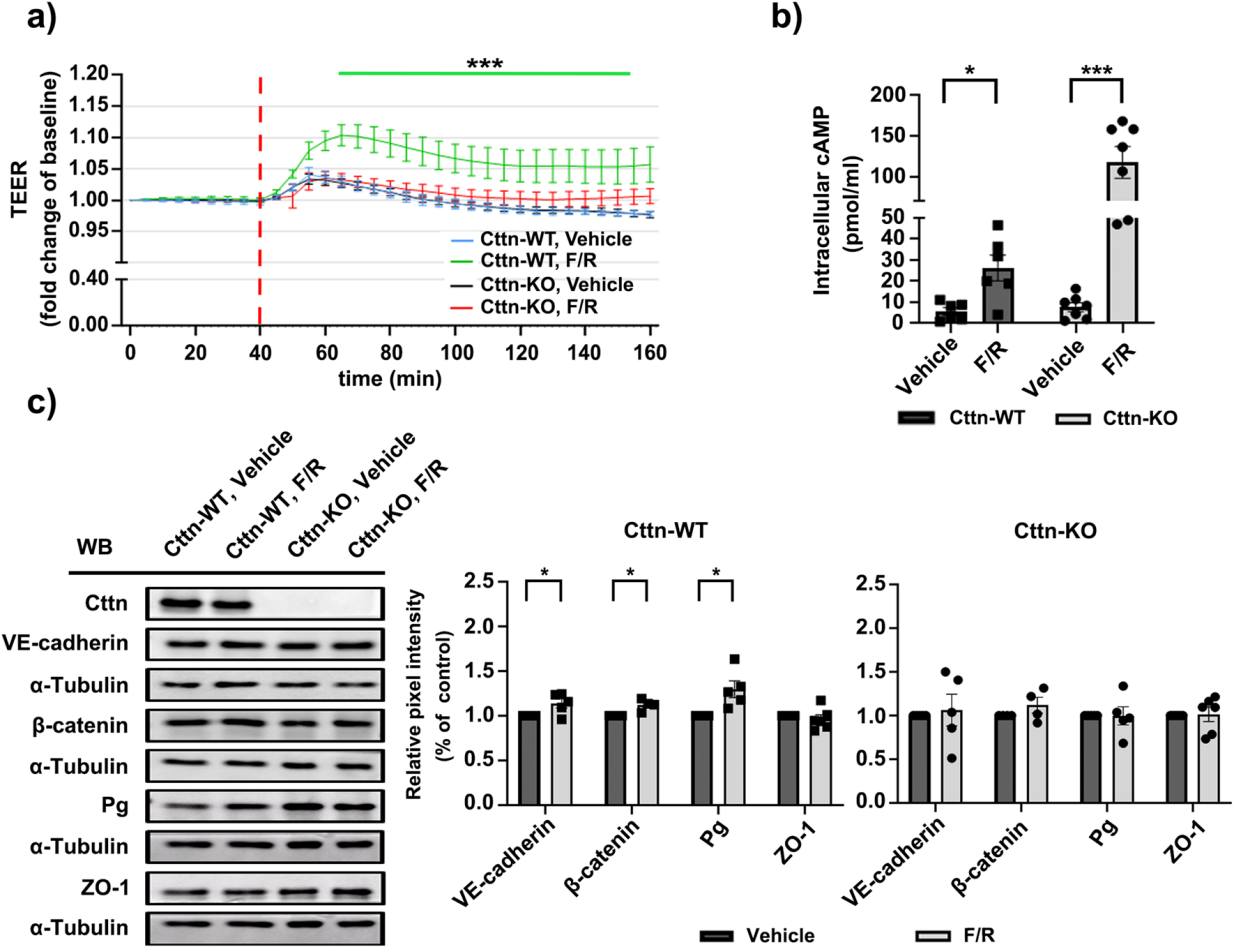
Barrier dynamics and protein expression upon cAMP elevation. (**a**) TEER measurements from confluent WT and Cttn-KO monolayers treated with either vehicle or F/R. The segmented red line indicates treatment application. (***) indicates the meaningful difference in TEER between WT and Cttn-KO cells after F/R application, N = 3 per group. (**b**) Bar diagram represents the intracellular cAMP concentration assessed by ELISA, N = 7 per group. (**c**) Western blot from confluent monolayers of WT and Cttn-KO cells treated with vehicle or F/R. Equal loading was verified by α-tubulin expression. The bar diagram represents relative expression of each protein of interest. Here, the densitometric measurements for each band of target protein was normalized to the corresponding control, N = 4–6. Data are represented as mean ± SEM; *p < 0.05; ***p < 0.001.

**Figure 5:**
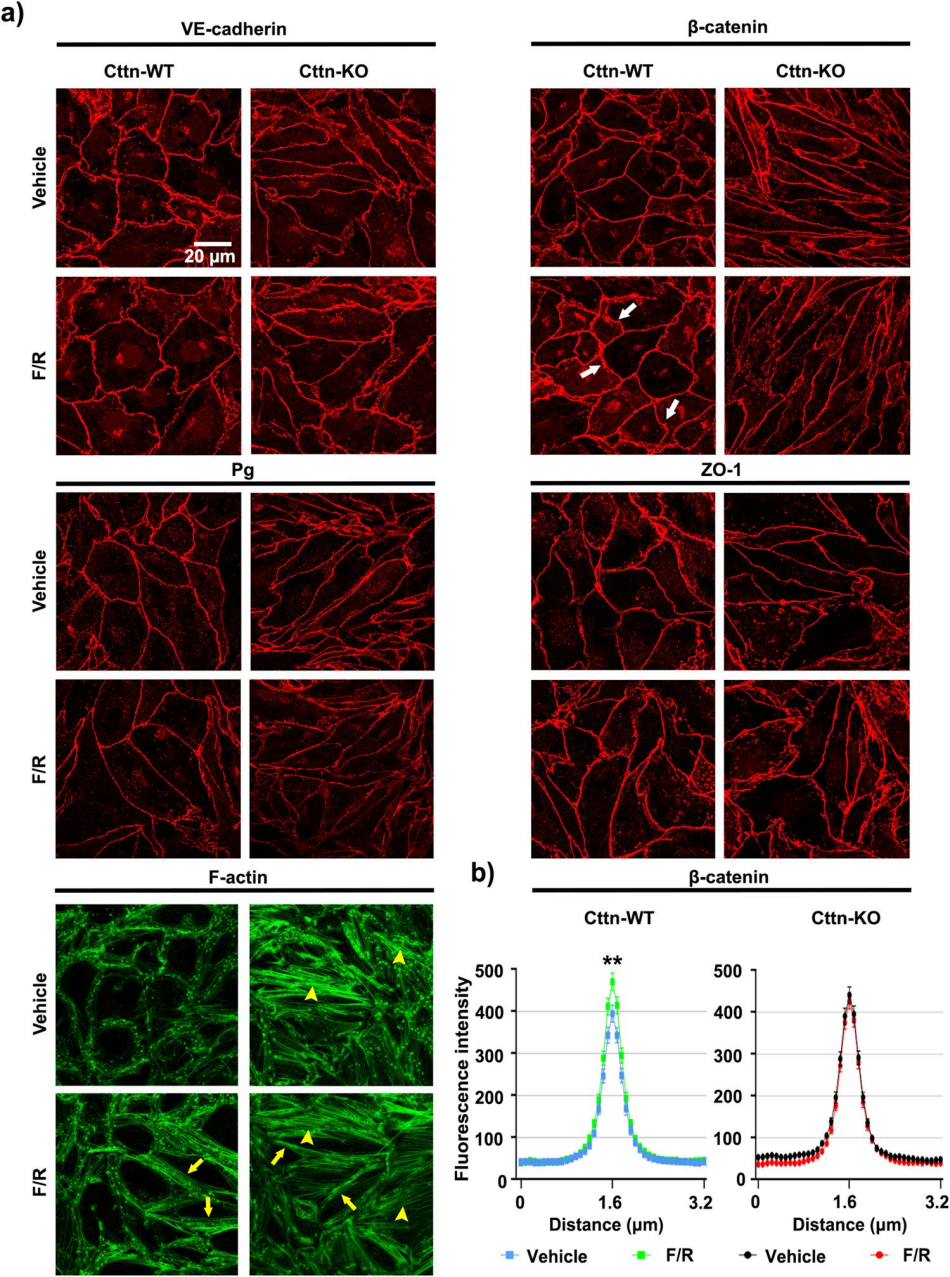
Localization of junctional molecules after F/R application. (**a**) Representative immunostaining for VE- cadherin, β- catenin, Pg, ZO-1 and F-actin in WT and Cttn-KO cells subjected to vehicle or mediator. White arrows show thicker areas of immunofluorescence signal. Yellow arrows illustrate cortical actin. Yellow arrowheads show the presence of intracellular actin fibers, N = 3–7. (**b**) Bell shaped curve represents the pixel intensity of β-catenin after Vehicle or F/R treatment in WT and Cttn-KO cells, N = 7. Data are represented as mean ± SEM; *p < 0.05.

It is widely accepted that augmented intracellular cAMP concentration benefits the endothelial barrier^40–43^. Although Cttn has been linked to cAMP-mediated signaling^26,38^ its role in the cAMP-dependent barrier stabilization or cAMP production is unclear. For this reason, we performed TEER measurements in confluent endothelial monolayers subjected to either vehicle (DMSO) or Forskolin and Rolipram (F/R), which act as an adenylyl cyclase activator/protein kinase A agonist and as a selective phosphodiesterase 4 inhibitor respectively. As expected, WT endothelial cells responded with increased TEER shortly after F/R treatment (Fig. 4a, green line). On the other hand, Cttn-KO cells did not respond to this stimulus (Fig. 4a, red line). Vehicle-treated cells displayed insignificant change during the experiment (Fig. 4a, blue line for WT; black line for KO). Additionally, we investigated the contribution of protein kinase A (PKA) by treating the cells with dihydrochloride (H89), a specific PKA inhibitor. Both WT and Cttn-KO cells displayed significantly lower TEER shortly after PKA inhibition. Interestingly, the minimum resistance registered was still significantly lower in cells lacking Cttn (Supplementary Fig. 3a).We hypothesized that the lack of response seen in Cttn-KO cells following F/R application could be due to their inability to produce enough cAMP. To corroborate this idea, cAMP Enzyme-Linked Immunosorbent Assay (ELISA) was done. To our surprise, under control conditions, the cAMP concentration between WT and KO cells was comparable (Fig. 4b). Moreover, in both cell lines, a meaningful increase of intracellular cAMP after F/R application was observed (Fig. 4b). The value was however, much higher in cells without Cttn. These findings suggest that Cttn is involved in cAMP-mediated barrier enhancement and may regulate cAMP production once adenylyl cyclase is activated or phosphodiesterase-4 inhibited. Next, we studied the protein dynamics of different junctional proteins after cAMP elevation by Western blot and immunostaining. We observed that WT cells responded to F/R with a reproducible modest but notable increase in the protein level expression of the AJ components, VE-cadherin, β-catenin and Pg. Meanwhile, Cttn-KO cells displayed no substantial rise after the treatment (Fig. 4c). However, these changes in protein expression did not translate to differential membrane distribution of the same molecules, except β-catenin, where the elevation of cAMP resulted in stronger junctional signal (Fig. 5a, arrows and 5b bell-shape graph; data for the other proteins not shown). Nevertheless, in Cttn-KO cells all molecules tested remained unchanged despite the increase of cAMP (Fig. 5a). As next, we explored the effects of cAMP on cytoskeleton dynamics. These experiments revealed that treatment of WT cells with F/R induced thickening of the cortical actin belt (Fig. 5a, yellow arrows). In Cttn-depleted cells, F-actin distribution was also affected by F/R application. Here, in contrast to the vehicle treated cells, the cortical actin was broader and easily observable upon treatment (Fig. 5a, yellow arrows). Nonetheless, the presence of intracellular actin fibers was detected in both control and treated Cttn-KO cells (Fig. 5a, yellow arrowheads). Likewise, the influence of PKA towards junctional structural stability was examined. WT and Cttn-KO monolayers treated with H89 showed disrupted VE-cadherin signal at the membrane, although the effect was more visible in the latter (Supplementary Fig. 3b, arrows). For β-catenin and ZO-1, however, the effect was less obvious, suggesting that PKA inhibition acts mainly on VE-cadherin. In addition, WT cells shifted from a well-defined cortical F-actin distribution to stress fibers along the cells (Supplementary Fig. 3b, yellow arrows and arrowheads, respectively). Whereas cells lacking Cttn, which already had abundant stress fibers, exhibited a less organized F-actin network after the treatment (Supplementary Fig. 3b, arrowheads). Our findings suggest that Cttn is required to strengthen barrier function following cAMP-mediated signaling; on the one hand, by allowing AJ proteins level build up and on the other, by promoting β-catenin membrane accumulation. This process appears to require an important actin cytoskeleton rearrangement.

### cAMP-mediated activation of Rap1 and Rac1 small GTPases require Cttn

**Figure 6:**
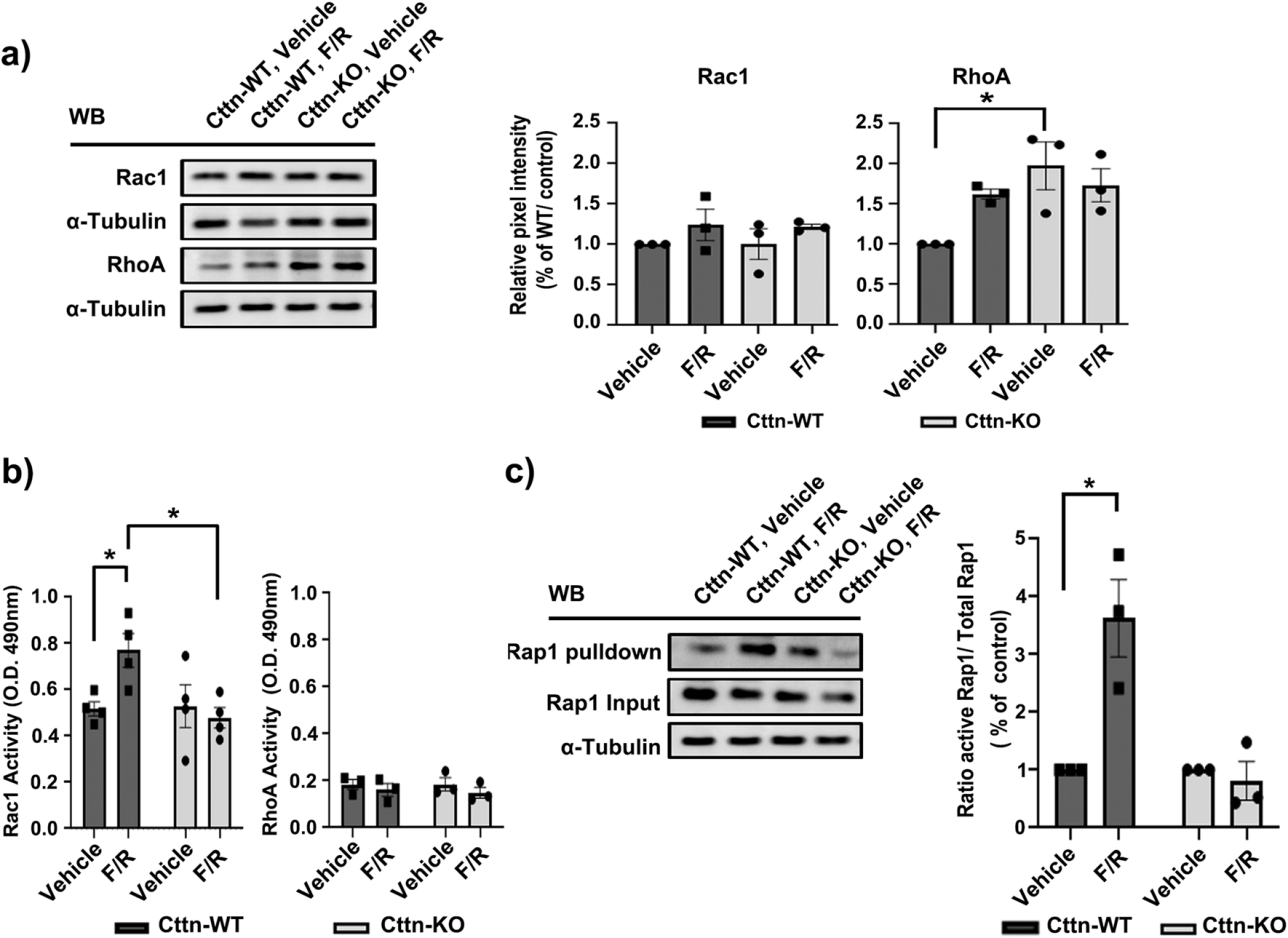
Analysis of small GTPases expression and activation after cAMP. (**a**) Protein level of small GTPases was analyzed after treatment with vehicle or F/R. Diagrams represent the densitometric band analysis normalized to WT vehicle, N = 3. (**b**) Rac1 and RhoA G-LISAs. The diagrams depict the level of small GTPases activity, before and after cAMP level elevation, N = 3–4. (**c**) Rap1 Activity analysis, the diagram represents the band intensity quantification, normalized to respective control, N = 3. Data are represented as mean ± SEM; *p < 0.05.

Since Cttn affected the cAMP-mediated barrier enhancement and previous studies have shown that it regulates small Rho GTPases^44,45^, we explored the contributions of Cttn towards cAMP modulation of Rap1, Rac1 and RhoA, well-known critical players in endothelial barrier homeostasis^46–48^. First, we investigated whether lack of Cttn affects the raw protein levels of the small GTPases Rac1 and RhoA. We found that the total amount of Rac1 was similar between WT and Cttn-KO cells in all experimental conditions tested. However, the expression of RhoA was substantially higher in vehicle-treated Cttn-KO compared to WT cells (Fig. 6a). Subsequently, we measured the activity of both GTPases by G-LISA. Our data showed that vehicle treated WT and Cttn-KO cells had equivalent Rac1 activation (Fig. 6b). More importantly, stimulation of cAMP was able to increase Rac1 activation in WT but not in cells lacking Cttn (Fig. 6b). In addition, despite the increased RhoA protein expression in Cttn-KO cells, the activity level was similar in both cell types with and without F/R (Fig. 6b). Finally, we evaluated the activation state of Rap1, known to function upstream of Rac1 and RhoA^5,49–51^. Notably, once cAMP increase was induced, only the WT endothelial cells reacted with considerably higher activation of Rap1 (Fig. 6c). These findings indicate that Cttn plays a role downstream of cAMP but upstream of Rap1.

### Synchronous activation of Rac1 and RhoA by CN04 lead to enhanced barrier function despite the absence of Cttn

**Figure 7:**
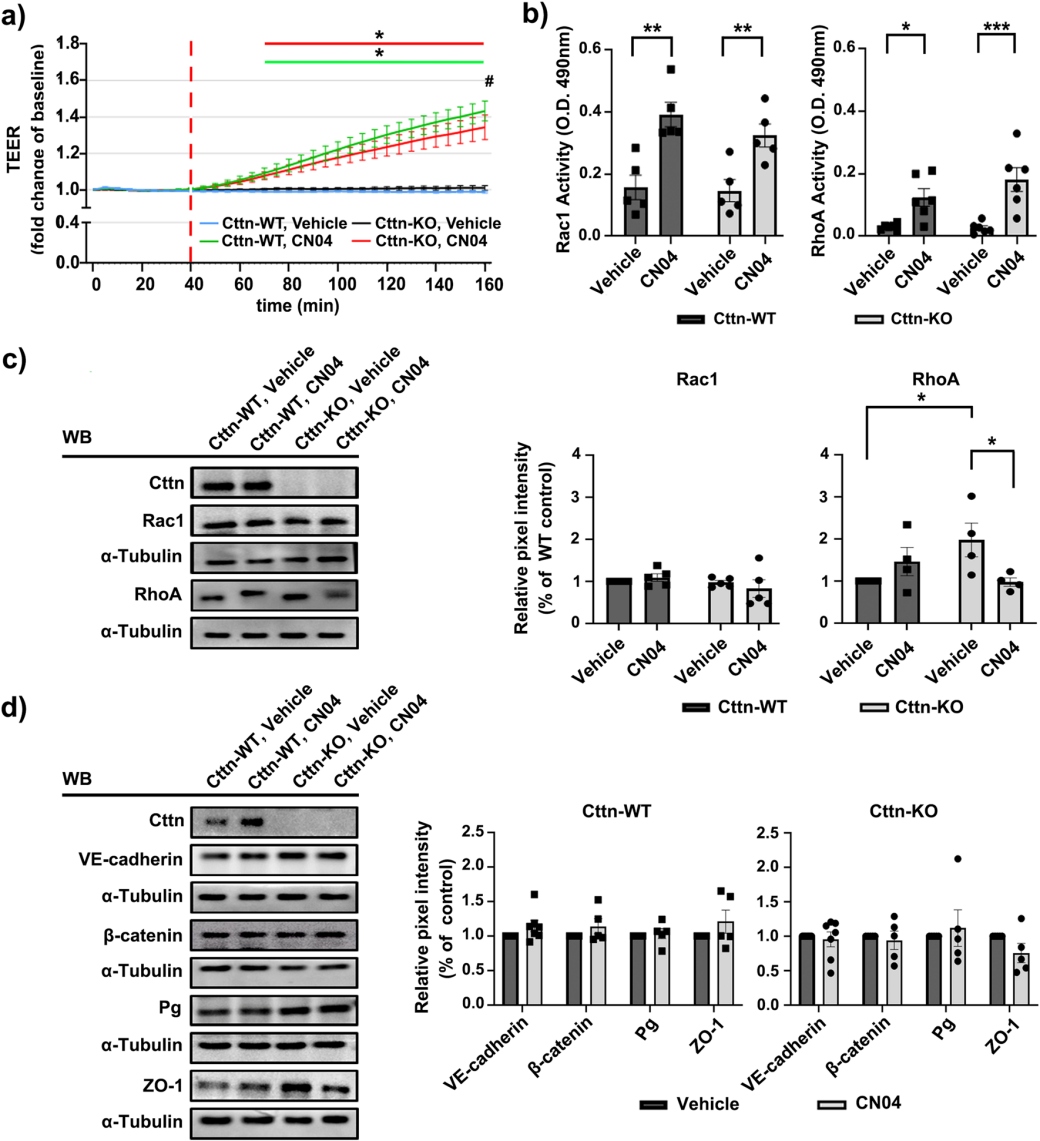
WT and Cttn-KO cells response to CN04 treatment. (**a**) TEER measurement of confluent monolayer treated with either vehicle or CN04. The diagram illustrates the recorded data normalized to the initial point. The segmented red line determines the start point of the treatment. (*) shows that CN04 treatment considerably improved barrier resistance in both cell lines. (#) Illustrates a substantial change between WT and Cttn-KO cells after CN04 treatment at the experimental end-point, N = 3. (**b**) G-LISA analysis for Rac1 and RhoA depicted in bar diagrams respectively, N = 4–5. (**c**) Representative Western blot for Rac1 and RhoA from WT and Cttn-KO cell monolayers treated with vehicle or mediator and the respective bar diagrams where the band intensity quantification was normalized to WT control, N = 4–5. (**d**) Western blot analysis of junctional proteins before and after CN04 treatment. The diagrams illustrate the densitometric analysis, normalized to the respective control, N = 4–7. Data are represented as mean ± SEM; *p < 0.05; **p < 0.01; ***p < 0.001.

**Figure 8:**
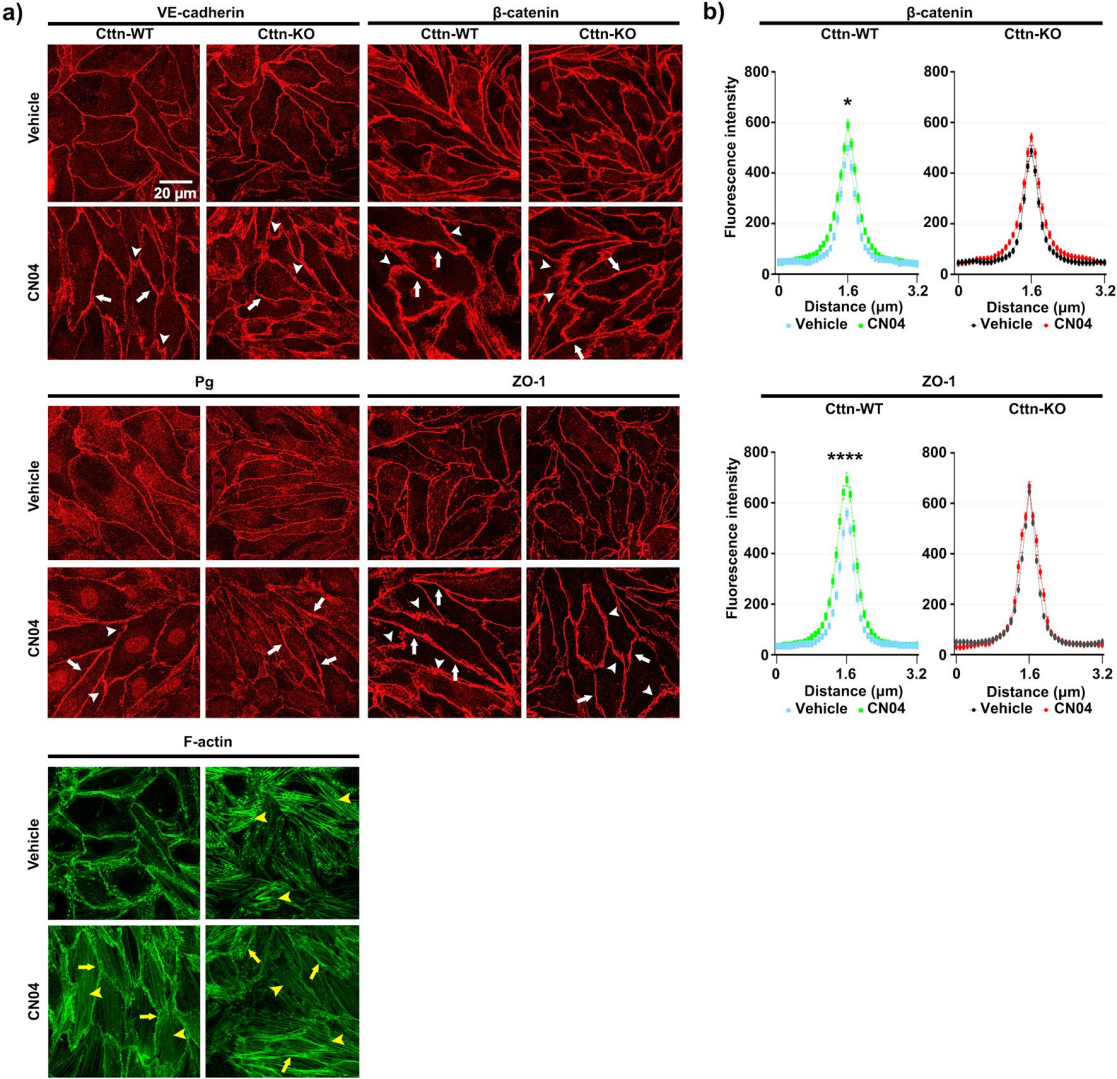
Junctional proteins localization and F-actin dynamics after simultaneous Rac1 and RhoA activation by CN04. (**a**) Immunostaining of junctional proteins distribution after treatment with vehicle or mediator. White arrows determine an increase in intensity and thickness, white arrowheads illustrate finger-like signals. Yellow arrows show cortical actin and yellow arrowheads display the presence of intracellular F-actin, N = 4–5. (**b**) The bell shape diagrams represent the densitometric measurement of the fluorescence signal for each protein of interest, N = 4–5. Data are represented as mean ± SEM; *p < 0.05.

As lack of Cttn affected both Rap1 and Rac1-mediated cAMP activation, we questioned whether Cttn may act downstream from small Rho GTPases independently of cAMP. To answer this, we treated the cells with CN04 which triggers direct simultaneous activation of Rac1 and RhoA. TEER measurements of confluent cell monolayers revealed that CN04 meaningfully enhanced the resistance in both cell lines (Fig. 7a, green line for WT; red line for KO). However, 2 h after the application, the increase was considerably higher in WT compared to Cttn-KO cells (Fig. 7A, “#”). Additionally, the activation of both GTPases was assessed by G-LISA. In line with the TEER data, the activity of Rac1 and RhoA was importantly increased following CN04 treatment and the effect was similar between WT and Cttn-KO cells (Fig. 7b). The total protein levels of Rac1 were also similar in both cell types, but as observed before, RhoA was upregulated in KO cells under control conditions. Interestingly, CN04 treatment attenuated RhoA expression in Cttn-KO cells (Fig. 7c). The data shows that Cttn is not required for cAMP-independent activation of Rac1 and RhoA. To understand further, how CN04 is able to augment endothelial barrier function, we analyzed the expression and localization of junctional molecules as well as the actin cytoskeleton. We observed that despite the strong increase in TEER caused by the treatment, there were no important changes in the total protein amount of VE-cadherin, β-catenin, Pg or ZO-1 neither in WT nor in Cttn-KO cells (Fig. 7d). Immunostainings for the same proteins revealed that CN04 application drives membrane mobilization of all proteins in both cell types, which was made evident by areas with slightly thicker and finger-like signal (Fig. 8a, arrows and arrowheads, respectively). However, quantification of the mean fluorescence intensity from all proteins along the membrane revealed no prominent change after CN04 (data not shown), except for β-catenin and ZO-1 where it was considerably higher in WT cells (Fig. 8b). On the other hand, while vehicle-treated WT cells displayed well-defined cortical F-actin distribution in both conditions (Fig. 8a); Cttn-KO endothelium had more actin fibers crossing the cell bodies (Fig. 8a, yellow arrowheads). Importantly, CN04 induced a more defined cortical actin pattern and increased amount of aligned intracellular actin fibers in WT cells (Fig. 8a, yellow arrow and arrowheads, respectively). In Cttn-KO cells, the cytoskeleton shifted to a better-defined cortical actin, similar to the control cells but not as strongly demarcated (Fig. 8a, yellow arrows and arrowheads, respectively). To differentiate the role of Rac1 and RhoA, we treated the cells with calpeptin (CN01), a specific RhoA activator. To no surprise, the treatment led to diminished barrier function of both WT and Cttn-KO cells. Nevertheless, the maximum resistance drop observed, was significantly lower in cells without Cttn (Supplementary Fig. 4a). Activation of RhoA after CN01 showed comparable behavior between both cell lines (Supplementary Fig. 4b). This effect was associated in part, with junctional fragmentation and formation of zipper-like VE-cadherin junctions (Supplementary Fig. 4c, white arrows and arrowheads, respectively), as well as with prominent presence of stress fibers (Supplementary Fig. 4c, yellow arrowheads). Thus, stimulation of RhoA alone, is detrimental for MyEnds’ barrier function. In summary, the data indicate that Cttn does not affect the cAMP-independent direct activation of Rac1 and RhoA. This activation is sufficient to augment barrier resistance even without the presence of Cttn, indicating that other proteins may compensate for the loss of this protein.

## Discussion

As a widely expressed multifunctional protein, Cttn was associated with diverse cellular functions ranging from synapse remodeling to cell migration and disease, such as pemphigus^52^ or cancer progression^53–58^, as well as cell junctions regulation^23,59^. However, the current information regarding the relationship between Cttn and endothelial cell–cell contacts is limited. It was documented that Cttn is important to preserve barrier function in different endothelial cells e.g., Cttn-deficient human dermal microvascular and human umbilical vein endothelial cells, as well as, in Cttn-KO murine lung endothelial cells (MLEC)^26^. In line with those findings, we show that Cttn is also critical for maintaining barrier function in myocardial endothelial cells. In our experiments, we found that the absence of Cttn resulted in a critical disruption of VE-cadherin, β-catenin and ZO-1 at cell junctions (Fig. 1). This is in contrast to the previous discovery where lack of Cttn did not affect VE-cadherin membrane distribution^26^ and to the report by Schnoor et al., showing no meaningful VE-cadherin protein regulation in mouse lung lysates or staining in cremaster venules after Cttn gene disruption^38^. On the other hand, our results matched those from the study by Citalán-Madrid, in which the absence of Cttn disrupted the cell contact localization of ZO-1 in mouse colonic tissue. An explanation for the discrepancy above is that the endothelial cells used across these studies have a different organ or tissue origin, which leads to endothelial heterogeneity^60^. Despite the differences, our findings that Cttn absence leads to lower TEER and altered actin cytoskeleton distribution (Fig. 1) are consistent with the literature^26,38,61–63^. A direct interaction between Cttn and any of the endothelial junctional components could explain the effects observed in this study. In fact, we were able to demonstrate that VE-cadherin, β-catenin and Cttn form a molecular complex in our endothelial model (Fig. 3), which to the best of our knowledge was not confirmed before. In regards of protein levels, we could only detect an increased amount of β-catenin in cells devoid from Cttn (Fig. 2). However, the mechanism behind the upregulation of β-catenin remains to be explored, but it may occur as a result from de novo protein synthesis or increased protein half-life involving the canonical Wnt signaling pathway^64,65^. We investigated if the Cttn/VE-cadherin interaction accounts for any important functional role in endothelial barrier regulation by combining TEER measurements with calcium switch assays. These experiments showed that in contrast to WT cells, disruption of Cttn gene expression led to slower barrier recovery after Ca^2+^ switch and the resistance value did not recover back to that of the vehicle-treated Cttn-KO cells (Fig. 3). However, the VE-cadherin intracellular fluorescent signal between WT and Cttn-KO cells was similar. The data suggest that the efficient recovery of the endothelial barrier requires the presence of Cttn. On the other hand, the interaction between Cttn and VE-cadherin, which appears to participate in this process may not be the only player governing VE-cadherin´s cellular distribution. Thus, it is possible to reason that Cttn partially mediates VE-cadherin expression or localization. The process may involve the degradation or accumulation of VE-cadherin shortly after its translocation to the cytosol, mediated by different pathways e.g., VE-cadherin tyrosine phosphorylation and activation of the Rho/ROCK/MLC pathway, endosome/lysosome or the proteasome^66,67^. In fact, Cttn has not only been linked to endosome/lysosome regulation^68,69^ but also to the Rho/ROCK/MLC signalosome in endothelial cells^26,70^. Given the connection between Cttn and this pathway, it is also tempting to speculate that the calcium/calmodulin-dependent serine/threonine phosphatase “calcineurin” (also known as PP2B), could be important in this context, because of its very well-known activity on the myosin phosphatase target subunit 1 (MYPT1), which prevents myosin contractility^71,72^. However, whether Cttn is somehow connected or not to PP2B in endothelial cells remains to be elucidated. This possibility is supported by the study from Passaro et al.^73^, where PP2B was shown to affect the expression of Cttn in leukemic T cells. Although the differences observed here cannot be solely attributed to VE-cadherin modulation, it is broadly accepted that it is the most important adhesion molecule for the stability of endothelial junctions^67,74,75^. Hence, our data indicates that the interaction between Cttn and VE-cadherin is of physiological importance and could occur directly or indirectly, given the broad possibilities allowed by the many functional domains comprising Cttn’s protein structure.

In the current study, we also explored the contribution of Cttn to cAMP-mediated barrier enhancement and could induce a notable increase of cAMP concentration with F/R treatment in both cell lines, however, MyEnds lacking Cttn were not able to improve barrier resistance as the WT controls did (Fig. 4), clearly indicating that Cttn is necessary for the cAMP-mediated barrier enhancement. Why are Cttn-KO cells unable to enhance barrier resistance after cAMP stimulation? This could be explained in part, by compartmentalization of the signaling molecule to different subcellular locations. Once translocated, it may require the contribution of additional signaling molecules which may include for example, the group of A-kinase anchor proteins (AKAPs) or IQ Motif Containing GTPase Activating Proteins (IQGAPs)^76,77^. With the current data, we can only speculate that such process requires the participation of Cttn. Our findings are in contrast to the study by Schnoor et al., where treatment of HUVECs or MLECs with the cAMP analogue 8-pCPT-2Me-cAMP (8-(4-Chlorophenylthio)-2’-O-methyl-cAMP; or 007) counteracted the increased permeability caused by Cttn knockdown^38^. This highlights that although different endothelial cell types share similar structure and molecular composition, the same signaling pathway may have an alternate modus operandi. In addition, cAMP induction led to a mild but noteworthy increase in the total protein level of junctional proteins but accumulation at the membrane was only visible for β-catenin (Fig. 4–5). An explanation for these effects could be that F/R triggers a rapid increase in barrier resistance starting with β-catenin mobilization. Indeed, different studies performed in models of blood–brain barrier and human dermal microvascular endothelial cells have demonstrated a critical role for β-catenin in vascular permeability and barrier-related genes regulation^78–80^. Although Cttn-KO cells had less defined cortical actin and more intracellular actin fibers (Fig. 1), we observed a considerable reorganization of the actin cytoskeleton following the increase of cAMP in both cell lines (Fig. 5). Therefore, our data suggest that this rearrangement supports junctional molecules redistribution to the membrane, but it is not entirely dependent on Cttn. In summary, our findings hint that β-catenin accumulation at the membrane may require the contribution of Cttn and its capabilities to serve as a molecular scaffold that enhances junctional proteins mobilization. This process could involve the actin cytoskeleton and related signaling proteins.

We analyzed the impact of PKA by treating the cells with H89. Our data showed that inhibition of PKA significantly decreases the barrier resistance of both WT and Cttn-KO cells. However, the values from WT cells were higher than those from cells lacking Cttn (Supplementary Fig. 3), implying a link between Cttn and PKA regulation. To the best of our knowledge, a direct interaction relating these molecules has not been reported. Nevertheless, different studies point towards this possibility. For example, Rezaee et al.^81^, demonstrated that application of forskolin to confluent airway epithelial cells activates PKA and prevents the accumulation of apical actin bundles and the translocation of Cttn to them, phenomena normally induced by the respiratory syncytial virus infection. Moreover, PKA was shown to co-distribute to invadopodia with the tumor-associated carbonic anhydrase IX (CAIX) and Cttn. In this context, however, activation of CAIX by PKA favors invadopodia and metastasis through a signaling pathway involving Cttn-cofilin, Arp2/3 complex and actin polymerization^82^. Thus, it is not clear whether the potential connection between PKA and Cttn is biologically positive or negative in regards to Cttn function, especially in endothelial cells.

We explored the role of the small GTPases Rac1 and RhoA, which have well characterized functions on cytoskeletal and cell contact dynamics^46,48^. We found that the activation state of both signaling molecules under resting conditions was comparable between the cell lines analyzed, suggesting that Cttn does not play a role in controlling Rac1 or RhoA activity and probably acts downstream of these molecules. On the other side, increased cAMP concentration by F/R triggered Rac1 activation only in WT endothelium and had no effect on RhoA activity in either WT or KO cells. Furthermore, Rap1 activation was hindered in cells lacking Cttn (Fig. 6). Combined, our data demonstrate a clear contribution of Cttn towards the cAMP-mediated activation of the Rap1/Rac1 axis to engage endothelial barrier strengthening, an idea which has been suggested by previous reports^26,38^ but not proven yet. In this regard, we have reported that Epac1 has a more prominent role than PKA in MyEnds^83^. This idea is supported by our finding that WT cells could elevate barrier resistance after F/R or 007 treatment, but not after specific activation of PKA by 6-Bnz-cAMP. Thus, it is tempting to hypothesize that Cttn collaborates with Epac1 or different GTPase-activating proteins (GAPs) or guanine nucleotide exchange factors (GEFs) to drive cAMP signaling and improve endothelial barrier function. For instance, it was shown that Cttn through its SH3 domain, is able to interact with the RhoGAP known as BPGAP1 to target Cttn towards the cell periphery and support cell migration^84^. Additionally, the RhoAGAP p190RhoGAP binds to Cttn via the protrusion localization sequence (PLS) and regulates the activity of RhoA^85^. Nevertheless, no evidence has been provided approaching the intricate nature of such regulations in regards to endothelial barrier homeostasis and the cAMP-mediated signaling pathway.

Simultaneous activation of small Rho GTPases by CN04 resulted in a substantial increase in TEER of both cell lines, bypassing the absence of Cttn (Fig. 7). This effect was accompanied by slightly improved membrane localization of junctional proteins in both WT and Cttn-KO cells. The cytoskeleton was also altered by CN04, where a more defined cortical actin belt in both cell lines was visible. Although, these effects were not as prominent in cells lacking Cttn, they seemed to be enough to develop a stronger endothelial barrier. These results show that other proteins can compensate for the lack of Cttn and support cytoskeletal and junctional reorganization following small GTPases activation. In contrast, activation of RhoA alone weakened barrier function regardless of the presence or absence of Cttn, but the absolute resistance values were significantly lower in the KO cells. This was a consequence of altered VE-cadherin junctional distribution and enhanced presence of actin stress fibers (Supplementary Fig. 4). These results are not surprising, since it is widely accepted that RhoA induces endothelial barrier disruption^86–88^. Thus, our data highlights two possibilities: (a) the barrier-fortifying effects caused by CN04 treatment require a coordinated and well-tuned spatial and temporal regulation of Rac1 and RhoA; or (b) the effects seen are predominantly mediated by Rac1 activation, as reported by others^89–91^. A cooperation between Rac1 and RhoA in corneal endothelial cells was exposed by Ortega M. C. et al.^92^. This demonstrates that we still do not fully understand the complex molecular machinery involved in the regulation of these GTPases and that it requires further investigation.

In summary, our data set a precedent to place Cttn as a molecular hub able to interact with different junctional proteins like VE-cadherin and β-catenin to preserve basal endothelial junction stability and on the other hand, support cAMP-mediated barrier fortification by enabling the activation of Rap1 and Rac1. Finally, it is important to note that the functions of Cttn in modulation of cAMP-mediated regulation of small GTPases is in line with previous findings on vasodilator-stimulated phosphoprotein (VASP)^93,94^ and adducin^13^. From this, we conclude that a central function of actin binding proteins associated with endothelial AJ is to fine-tune small GTPases such as Rap1, Rac1 and RhoA in response to changes in cAMP levels during inflammation and potentially also to extracellular mechanical cues.

## Material and methods

### Endothelial cells isolation and ethical approval

The transgenic C57BL/6J Cttn-mice were provided by Prof. Klemens Rottner (Department of Molecular Cell Biology, TU Braunschweig). Cttn-deficient animals do not show any visible phenotype, are healthy and have a normal life expectancy^38,52^. The animals were hosted in our local animal facility. The handling and use of the mice were approved by the Ethics committee of the Regierung von Oberbayern, Germany (Gz. 55.2-1-54-2532-139-2014). This study is in accordance with ARRIVE guidelines (https://arriveguidelines.org). All methods were performed in accordance with the relevant guidelines and regulations. The litters were generated by crossing Cttn-Heterozygous adult mice. Dr. Mariya Y. Radeva was aware about the mice allocation and handling; she performed the isolation of the cells. A total number of 9 neonatal mice (2–4 days old) derived from 1 litter were used for Myocardial Endothelial cells (MyEnds) isolation. The WT and Cttn-KO cell lines analyzed in the current project are derived from a single animal. The pups were transferred to the animal trials room inside a thermo-isolated dark box filled with soft padding to reduce exposure to light, noise and sudden temperature alterations. Once in the room, the pups were left to acclimate for at least 30 min inside the box. One by one, the mice were transferred to a sterile S2-laminal flow hood and were sacrificed by decapitation without anesthesia. Next, the tip of the tail was dissected and processed for DNA extraction and genotyping. Subsequently, the heart was extracted and MyEnds were isolated as described previously^13,43^. Briefly, the isolation procedure was performed at room temperature under the laminar flow hood. Here, the mouse myocardial tissues were chopped into small fragments, which subsequently were digested with Trypsin (0.05%)-Collagenase A (0.02%) solution for at least 2 h at 37 °C, receiving vigorous shaking every 15 min. The digestion was terminated by adding an equal volume of ice-cold buffer (153 mM NaCl, 5.6 mM KCl, 2.3 mM CaCl_2_ × 2H_2_O, 2.6 mM MgCl_2_ × 6H_2_O, 15 mM HEPES, 1% BSA) to the tissue debris and cell suspension. After short centrifugation, the supernatant was gently removed and the resulting pellet was resuspended into the Dulbecco’s Modified Eagle’s medium (DMEM, Gibco-Thermo Fisher, #41966-029), supplemented with 50 U/ml Penicillin G/Streptomycin (Sigma Aldrich Chemie GmbH, Taufkirchen, Germany), and 10% Fetal calf serum (FCS, Biochrom, #S0115/0247X). Cell suspensions were cultivated on gelatin-coated dishes and grown in humidified incubator at 37 °C with 5% CO_2_. One day after plating, adherent cells were transfected with Polyoma virus middle T antigen secreted by GP+E-86 Neo (GPENeo) fibroblast. This treatment causes growth advantage of endothelial over nonendothelial cells, resulting to a homogeneous monolayer of endothelial cells after 4–6 weeks of culturing. The endothelial markers von Wilebrand Factor (vWF), PECAM-1 and VE-cadherin were used to confirm the cells´ phenotype. In addition to PCR genotyping, Western Blot and immunostaining were conducted to confirm the presence or lack of Cttn in the cells (Supplementary Fig. 1). All experiments were performed in vitro.

### Test reagents and antibodies

To elevate intracellular cAMP concentration, 5 μM Forskolin (Sigma Aldrich Chemie, #F6886), and 10 μM Rolipram (Sigma Aldrich Chemie, #R6520) were applied for 1 h. To stimulate the activation of Rho family small GTPases cells were treated with 0.25 μg/ml Rho/Rac/Cdc42 Activator I (Cytoskeleton Inc, #CN04-A) for 2 h. Triggering of RhoA was achieved with the Rho activator I, Calpeptin at 1 U/ml (#CN01-A, Cytoskeleton Inc.). Inhibition of PKA was achieved using 10 µM Dihydrochloride (H89, Santa Cruz, sc-3537).

The following antibodies were used in this study.

### Western blot

Cells were washed with ice-cold PBS and lysed with SDS-lysis buffer (25 mM HEPES, 2 mM EDTA, 25 mM NaF and 1% SDS, pH 7.6), in combination with cOmplete™ protease inhibitor cocktail (Roche Diagnostics, #11697498001) and PhosStop EASYpack (Roche Diagnostics, #4906845001). The resulting whole cell lysates were sonicated, centrifuged at 4 °C and 14,000 rpm for 1 min, right after the supernatants were collected and transferred to fresh tubes. Protein concentration was estimated by BCA standard colorimetric assay (Thermo Fischer Scientific, #23225). Next, samples were mixed 1:1 with 3 × Laemmli buffer and denatured by boiling for 5 min at 95 °C. Samples were separated by SDS-PAGE and transferred onto nitrocellulose membrane (Thermo Fischer Scientific, #LC2006), with 0.45 µm pore size. Membranes were blocked with 5% bovine serum albumin (BSA) in Tris-Buffered Saline with 0,1% Tween (TBS-T) for 1 h at room temperature and subsequently incubated with the primary antibodies of interest overnight at 4 °C on a rocker. The day after, the membranes were washed with TBS-T and incubated with horse-radish-peroxidase-(HRP)-species-specific secondary antibodies at room temperature for 1 h. After three rounds of washing with TBS-T, proteins were visualized using the Amersham Imager 600 (GE Healthcare, AI600). Pixel intensity quantifications from SDS-PAGE were performed using ImageJ (NIH, Windows version 64-bit).

### Immunoprecipitation

To conduct the experiment, cells were cultured in T75 flask and lysed with NP-40 lysis buffer (10 mM HEPES, pH 7.9, 1.5 mM MgCl_2_ × 6H_2_O, 10 mM KCl, 5 mM EDTA, 2 mM EGTA and 1% NP-40) containing cOmplete™ and PhosStop EASYpack. Samples were kept on ice for 30 min and passed through a 20G needle 10–12 times. Next, supernatants were collected after separation from the pellet by centrifugation at 10,000 rpm for 3 min at 4 °C. Once the protein concentration was estimated, 1000 µg of protein were mixed with lysis buffer to obtain a total volume of 1 ml. Next, samples were placed on rotator and incubated with pre-washed protein A/G agarose beads (Santa Cruz, #SC-2003) for 1.5 h at 4 °C in order to prevent non-specific binding. After centrifugation (15,000 rpm, 10 min, 4 °C), supernatants were mixed with either 1 µg of VE-cadherin Rabbit antibody or respective IgG control antibody (Cell Signaling Tech, #2729S) overnight on an overhead rotator at 4 °C. Next day, samples were transferred to pre-washed agarose beads and mixed on rotator for 2 h as explained above. Subsequently, beads were collected after centrifugation (15,000 rpm at 4 °C for 8 min), washed and mixed with 22 μl 1 × Laemmli and boiled for 10 min at 95 °C. Following a final centrifugation step at 15,000 rpm for 5 min at room temperature, 20 μl of each supernatant was loaded on SDS-PAGE and analyzed by Western blot as described before.

### Evaluation of the mRNA levels by PCR analysis

To analyze the mRNA level of junctional proteins, total RNA was extracted using RNeasy Plus Mini Kit (QIAGEN, #74134). cDNA was prepared using the SuperScript™ II Reverse Transcriptase (Thermo Fisher, #18064014) according to the manufacture’s instruction. PCR analyses were performed using following primers with the same conditions, initial denaturation for 3 min at 95 °C, 35 cycles of 30 s at 95 °C (denaturation), 30 s at 55°/60 °C (annealing) followed by 45 s at 72 °C (extension). The housekeeping gene ß2-microglobulin (B2M) was employed as a loading control. Pixel intensity quantifications from agarose gels were performed using ImageJ.

### Transendothelial electrical resistance (TEER) measurements

To monitor barrier integrity over time, TEER measurements were performed using the ECIS Z Theta system (Applied Biophysics). The 8W10E gold arrays (Ibidi, #72010) were stabilized and coated with gelatin, cells were seeded, electrodes were mounted on the ECIS station and the measurements were started. Every 5 min, electrical resistance was measured using the multi-frequency mode but the frequency of 4000 Hz was used for data presentation.

### Immunostaining

Immunofluorescence stainings were performed to analyze the localization of junctional proteins. For this purpose, MyEnd cells were seeded on 12 mm glass coverslip coated with 5% gelatin. Confluent monolayers were fixed with 4% paraformaldehyde for 10 min at room temperature. Membrane permeabilization was achieved using 0.1% Triton-X-100 diluted in PBS for 5 min. Unspecific antibody binding was blocked by incubating the cells with a mix of 1% bovine serum albumin (VWR, #422351S) and 10% normal goat serum (Thermo Fisher Scientific, # 31872) for 20 min. To detect the proteins of interest, cells were incubated with primary antibodies for 1 h at room temperature. After several washes with 1 × PBS, the monolayers were incubated with a mix solution containing species-specific Cy3-labelled secondary antibodies, Alexa Fluor® 488 Phalloidin (Molecular Probes\Life technologies, #A12379) and Dapi (Roche, #10236276001) which were diluted to 1:400 and 1:200 respectively. After washing with 1 × PBS and ddH2O, the coverslips were mounted on microscope glass slides with mounting media. The images were taken with a laser scanning confocal microscope (Leica SP5) equipped with a HCX PL APO Lambda blue 63 × 1.4 oil immersion objective (Leica).

### Quantification of junctional and intracellular pixel intensity

Confocal immunofluorescence full projections were analyzed with the ImageJ software^95^. To quantify the intensity and signal distribution of cell contact proteins, Z-stack full projections were prepared, the straight-line tool was used to draw a line that crosses the junctional signal perpendicularly. An equal number of points before and after the highest value was recorded to generate a bell-shape diagram. To quantify the intracellular pixel intensity the built-in “rectangle” tool was used. Rectangles were drawn on 5 random intracellular areas from at least 10 different cells per experiment and condition. To discard differences caused by the size of the line or square, the “integrated density” was recorded.

### Quantification of junctional fragmentation

Junctional fragmentation was quantified as previously described^13^. Briefly, after preparation of the images, the threshold value should be set to eliminate the unspecific staining, background. Next, using skeletonized 2D/3D tools, the junctional signal linearized and appeared with 1 pixel thickness. To quantify the fragmentation which has value 0, freehand tool was employed to manually draw a line along the junction. The total number of “0” values counted are divided by the total number of pixel values multiplied by 100 determines the percentage of fragmentation.

### Calcium switch assay

Endothelial barrier recovery was investigated using confluent cell monolayers as described before^13^. In short, cells seeded on 8WE10 gold arrays or on gelatin-coated glass coverslips were treated with 2.5 mM EGTA for 30 min. Afterwards, CaCl_2_ was added to the medium to a final concentration of 5 mM. The TEER was measured during the whole experiment.

### Determination of cyclic-AMP concentration

The intracellular concentration of cAMP was determined using a commercially available ELISA kit (Sigma Aldrich Chemie, #CA200-1KT). The experiment was performed according to manufacturer’s protocol. The absorbance measurements were done using a wavelength of 450 nm using the TECAN Infinite 200 PRO microplate reader (Tecan Deutschland GmbH).

### Rap1 Activity measurement

Rap1 activation was assessed using the non-radioactive Rap1 activation kit (Sigma-Aldrich, #17,321) following the manufacturer’s instructions. Briefly, Rap1-GTP was pulled down using Ral GDS-RBD agarose beads. Subsequently, equal amount of protein (100 µg) from control and treated cells was used for Western Blot analysis. The pixel intensity of the resulting bands was quantified and the ratio between total and active Rap1 was calculated.

### Colorimetric G-LISA for Rac1 and RhoA activity measurements

The activation state of small GTPases was estimated using the Rac1 and RhoA G-LISA kits (Cytoskeleton, #BK128 and #BK124, respectively). The experiments were conducted based on the manufacturer’s protocol and the absorbance measured at 490 nm using a TECAN Infinite 200 PRO device.

### Statistical analysis

To perform statistical analysis, Prism Software version 8 (Graph pad) was used. Data are presented as mean ± SEM. To statistically compare the difference between two groups, unpaired two-tailed Student T-test was applied and for three or more conditions, Two-way analysis of variance (ANOVA) followed by Sidak ´s multiple comparison test. Values equal or below 0.05 (*); 0.01 (**); 0.001 (***) and 0.0001 (****) were considered statistically significant.

### Supplementary Information

Supplementary Figures.

## Data Availability

The datasets generated and/or analyzed during the current study are available upon reasonable request to the corresponding author. We do not have the means to provide a permanent link to a storage database.

## Abbreviations

MyEnd: Myocardial Endothelial cells
ADM: Adrenomedullin
Epac1: Exchange Protein Directly Activated By CAMP 1
PKA: Protein Kinase A
Cttn: Cortactin
TJ: Tight junction
AJ: Adherens junction
Pg: Plakoglobin, Y-catenin
TEER: Transendothelial electrical resistance
cAMP: Cyclic adenosine monophosphate
IP: Immunoprecipitations
CN04: Rho/Rac/Cdc42 Activator I
CN01: Calpeptin
H89: Dihydrochloride
F/R: Forskolin/Rolipram
GTPase: Guanosine-Triphosphatase
ELISA: Enzyme-Linked Immunosorbent Assay
WT: Wild-type
KO: Knock-out
BSA: Bovin serum albumin
TBS-T: Tris-Buffered Saline with 0.1% Tween
SDS-PAGE: Sodium dodecyl-sulfate polyacrylamide gel electrophoresis
