# Supplementary material for: Cortactin is in a complex with VE-cadherin and is required for endothelial adherens junction stability through Rap1/Rac1 activation
Source: Sci Rep. 2024 Jan 12;14:1218. doi: 10.1038/s41598-024-51269-3 (PMC10786853; doi:10.1038/s41598-024-51269-3)
Supplement: Supplementary file 1 — Supplementary Figures. [file 41598_2024_51269_MOESM1_ESM.pdf]

Cortactin is in a complex with VE-cadherin and is required for endothelial adherens junction stability through Rap1/Rac1 activation

*Sina Moztarzadeh, Sara Sepic, Ibrahim Hamad, Jens Waschke, Mariya Y. Radeva and Alexander García-Ponce**

Chair of Vegetative Anatomy, Faculty of Medicine, Ludwig-Maximilians-University (LMU) Munich, Pettenkoferstraße 11, 80336, Munich, Germany.

**corresponding author*

Tel. +49 89 2180 - 72699Mail. alexander.garcia@med.uni-muenchen.de

Figure S1.

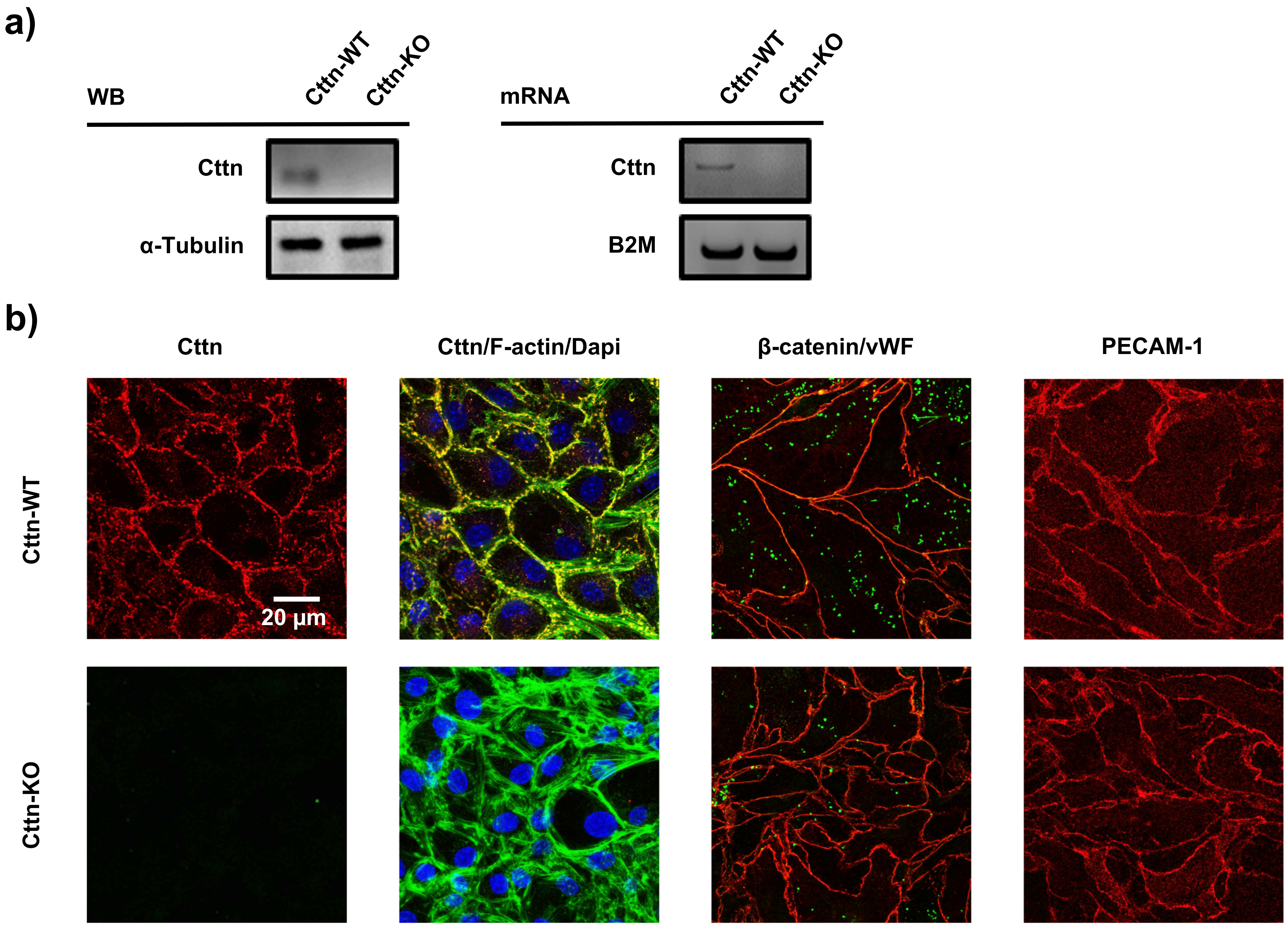

Figure S1. a) WB and PCR analyses demonstrating the ablation of Cttn protein and gene expression, N= 3. b) Immunostainings for Cttn and specific enodthelial lineage markers, N= 3.

Figure S2.

a)

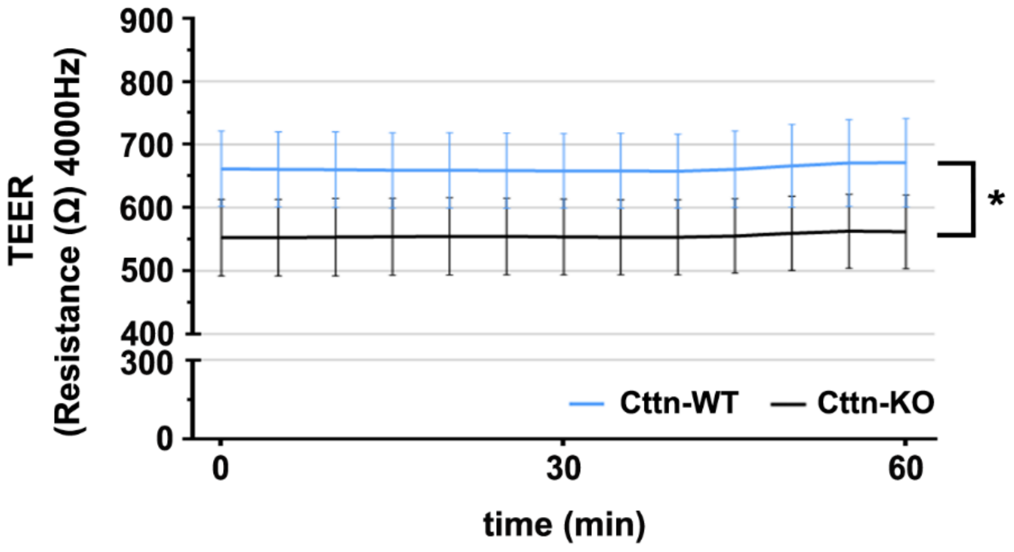

b)

	Rb	Alpha (α)
Cttn-WT	0.2171	2.811
Cttn-KO	0.06500	2.920

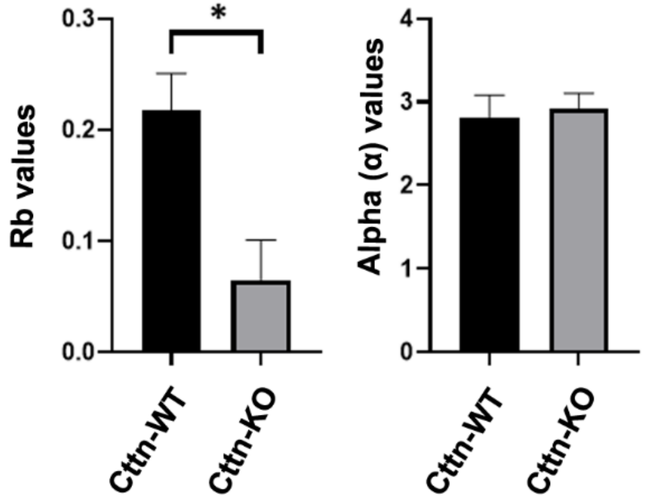

Figure S2. a) TEER raw values comparing the resistance between WT and Cttn-KO cells, N= 3 b) Cell tightness (Rb) and cell substrate attachment (α). (*) indicates a significant difference between Cttn-WT and Cttn-KO values, N= 3. Data are represented as mean ± SEM; *$p < 0.05$.

Figure S3.

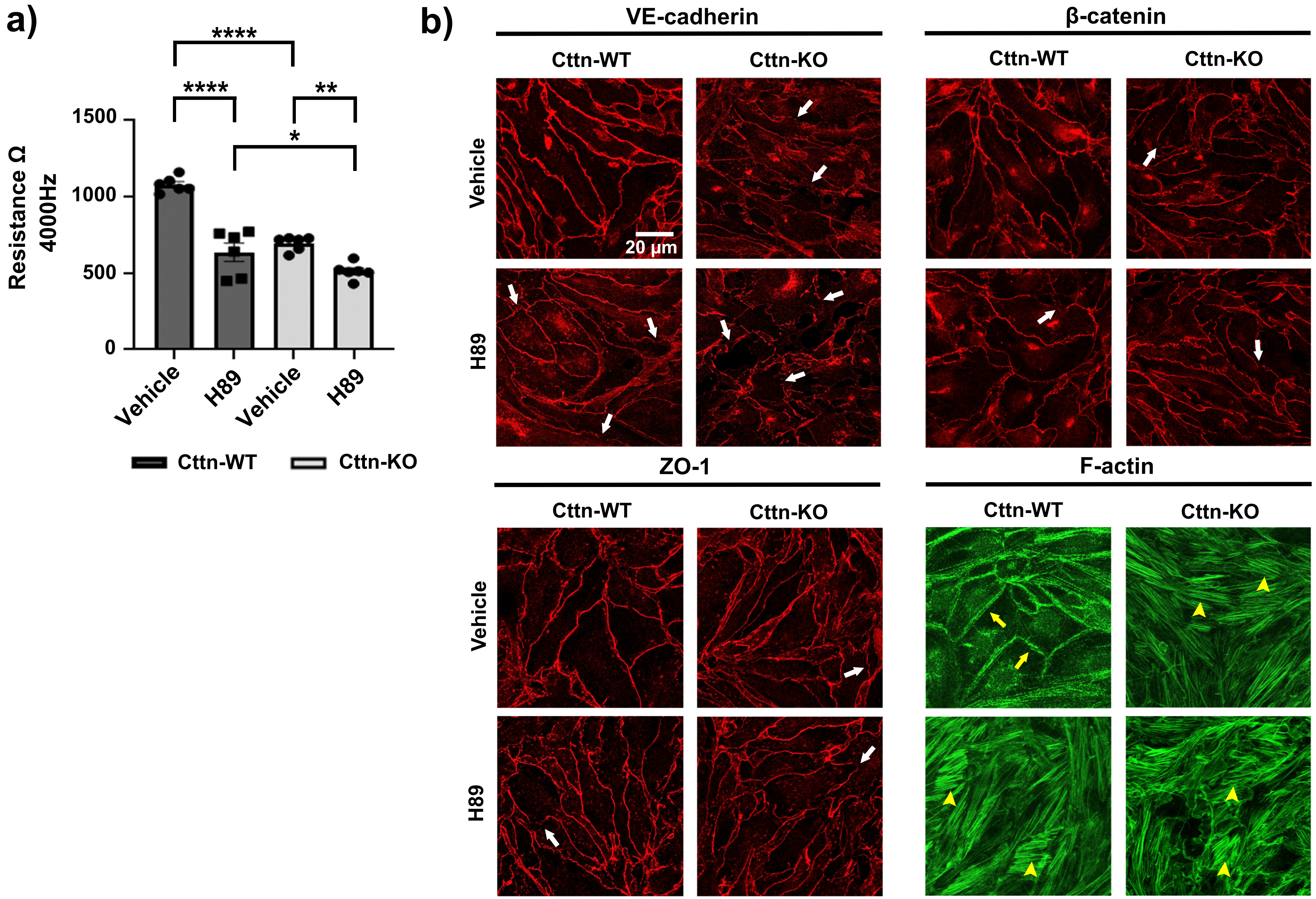

Figure S3. a) TEER measurements representing the resistance values for WT and Cttn-KO cells, 20 minutes after application of vehicle or H89. (*), (**) and (****) depict significant differences, N= 3. b) Immunostainings showing the distribution of VE-cadherin, β- catenin and ZO-1 from monolayers subjected to vehicle or mediator. White arrows indicate junctional fragmentation. Yellow arrows and arrowheads indicate cortical actin and stress fibers, respectively, N= 3. Data are represented as mean ± SEM; *$p < 0.05$, **$p < 0.01$; ***$p < 0.001$; ****$p < 0.0001$

Figure S4.

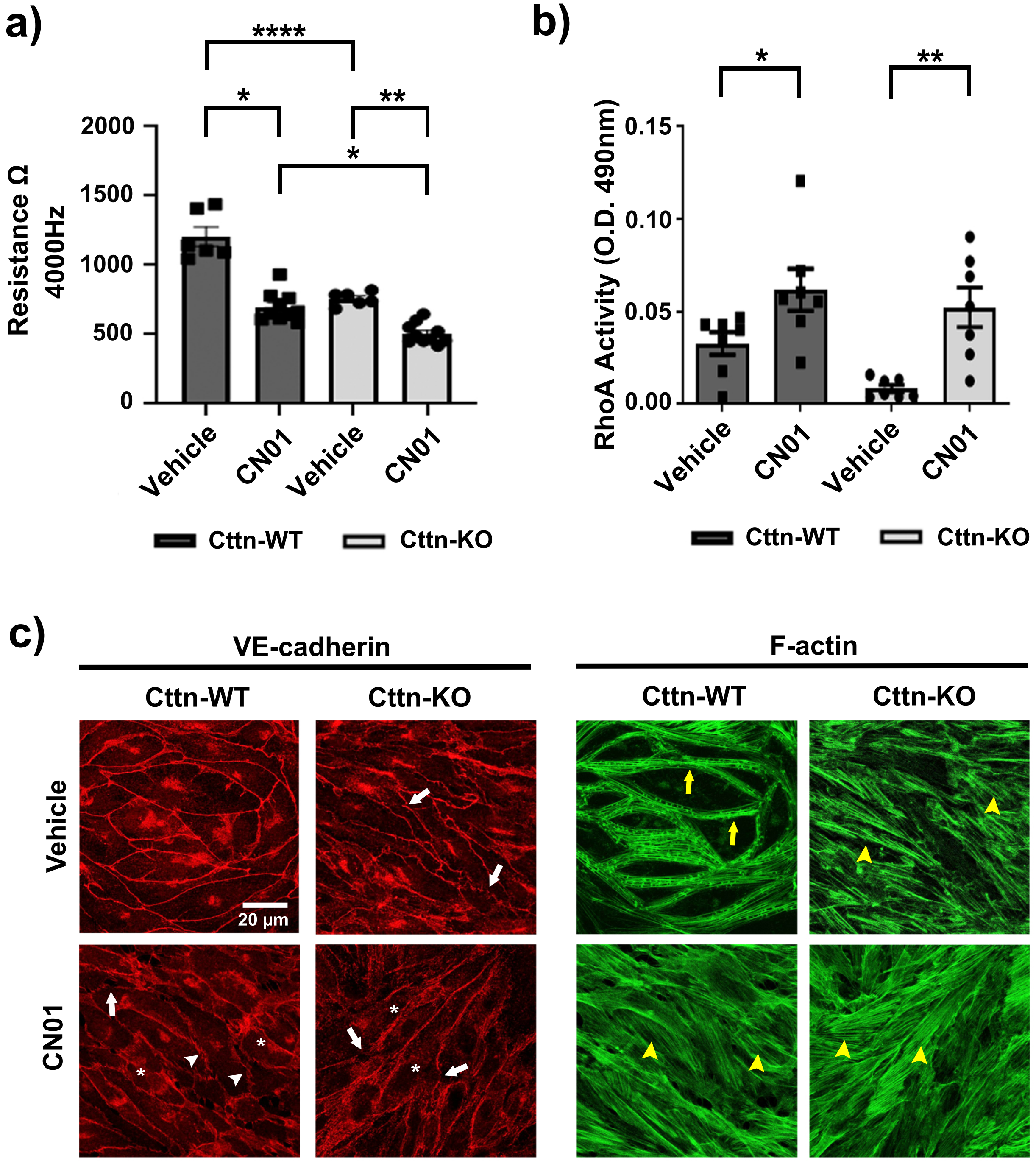

Figure S4. a) TEER measurements, the diagram represents the resistance values for WT and Cttn-KO cells treated with vehicle or CN01 after 20 minutes . (*), (**) and (****) indicate a notable changes, N= 3. b) RhoA activity following application of either vehicle or CN01 in confluent monolayers. (*), (**) indicate a significant activation of RhoA, N= 7. c) Immunostainings of VE-cadherin and F-actin. White arrows depict junctional fragmentation, asterisks represent VE-cadherin intracellular signal, white arrowheads portray zipper-like junctions. Yellow arrows and arrowheads indicate cortical actin and stress fibers, respectively, N= 3. Data are represented as mean ± SEM; $*p < 0.05$, $**p < 0.01$; $****p < 0.0001$

Original Western blot gel images for Basal condition

Figure S5, a)

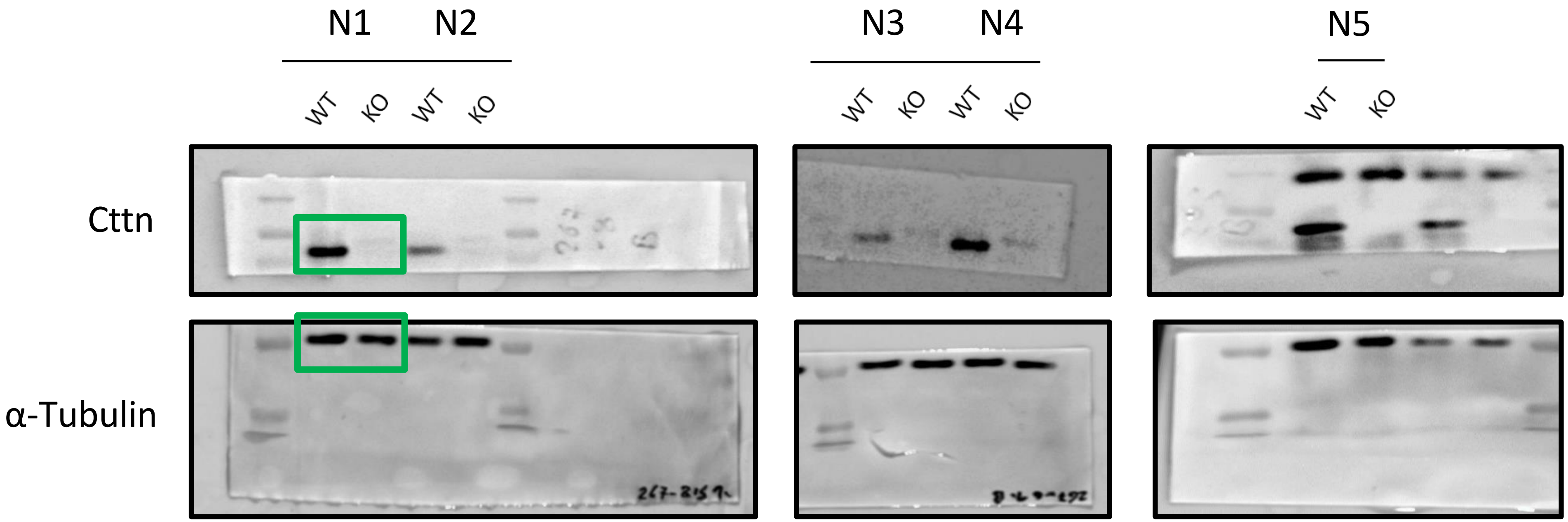

Figure S5, b)

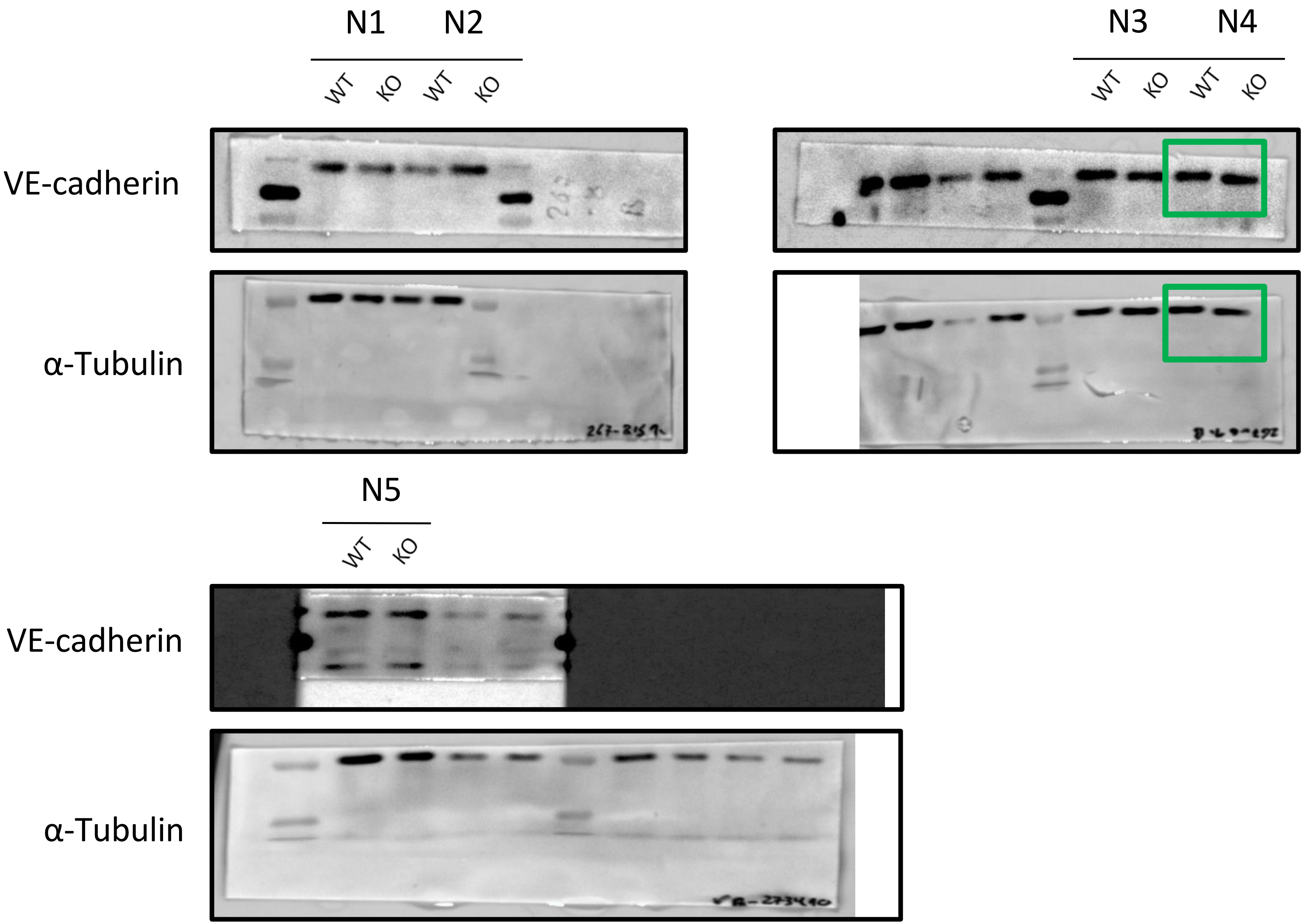
N1 N2
WT KO WT KO
N3 N4
WT KO WT KO
VE-cadherin
α-Tubulin
N5
WT KO
VE-cadherin
α-Tubulin

Figure S5, c)

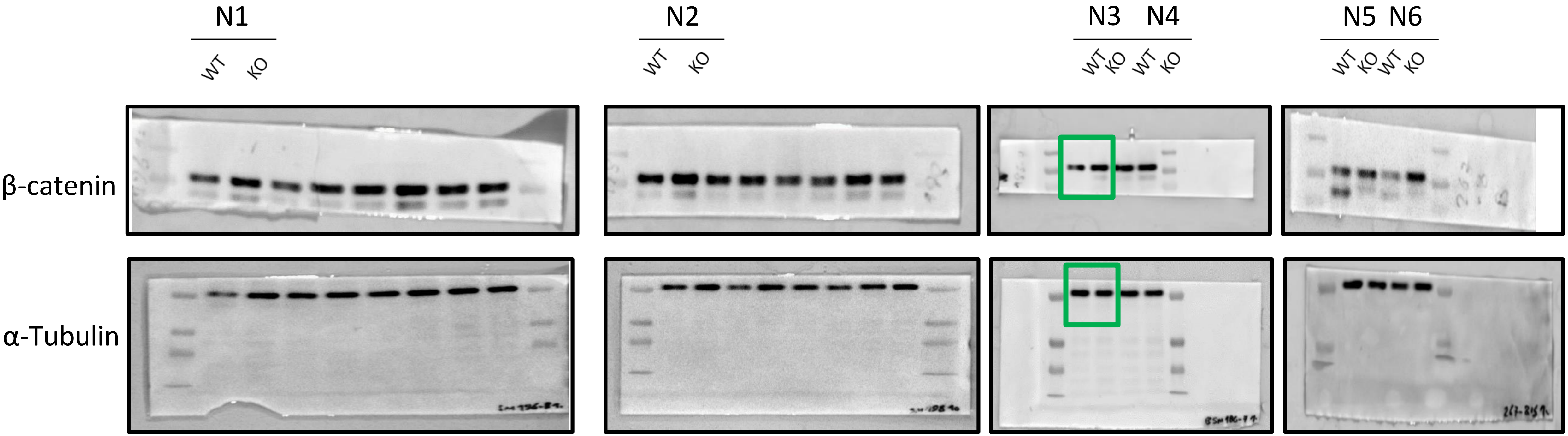
N1
WT
KO
N2
WT
KO
N3 N4
WT KO WT KO
N5 N6
WT KO WT KO
β-catenin
α-Tubulin

Figure S5, d)

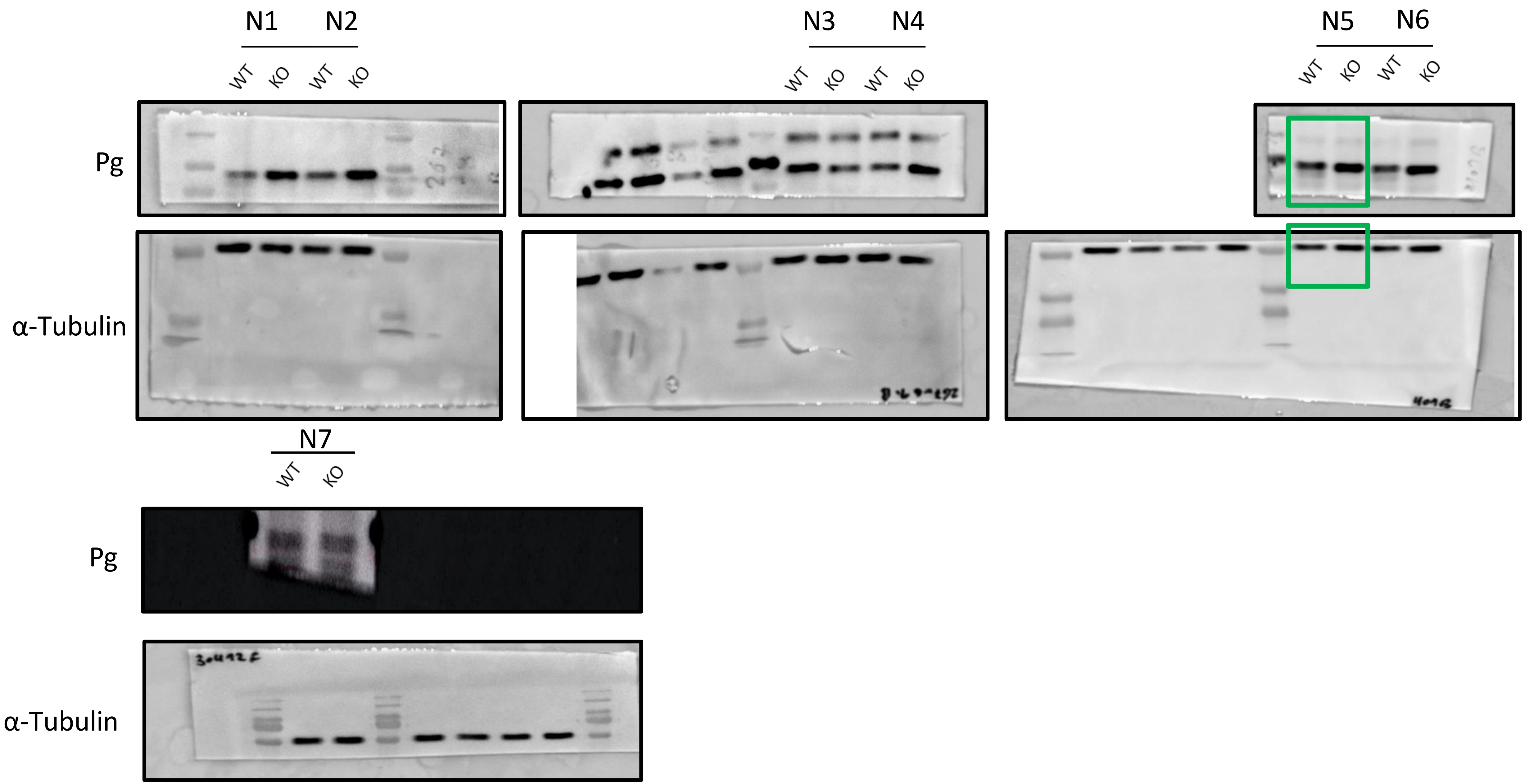

Figure S5, e)

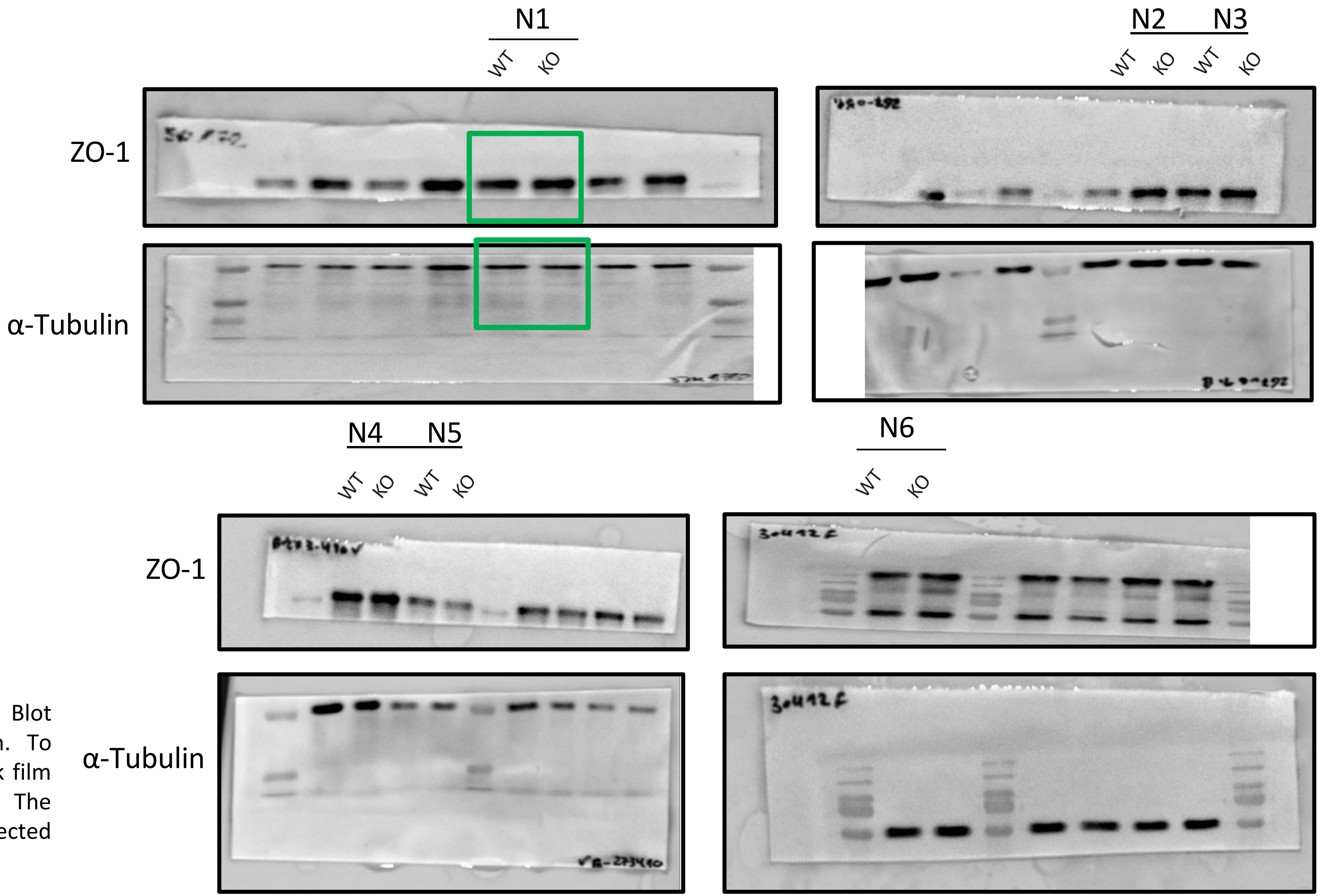

Figure S5, a-e) Original Western Blot gel images for basal condition. To prevent over exposure, the black film was used to cover the bands. The green square determines the selected blot used in the figure 2.

Original PCR gel images

Figure S6, a)

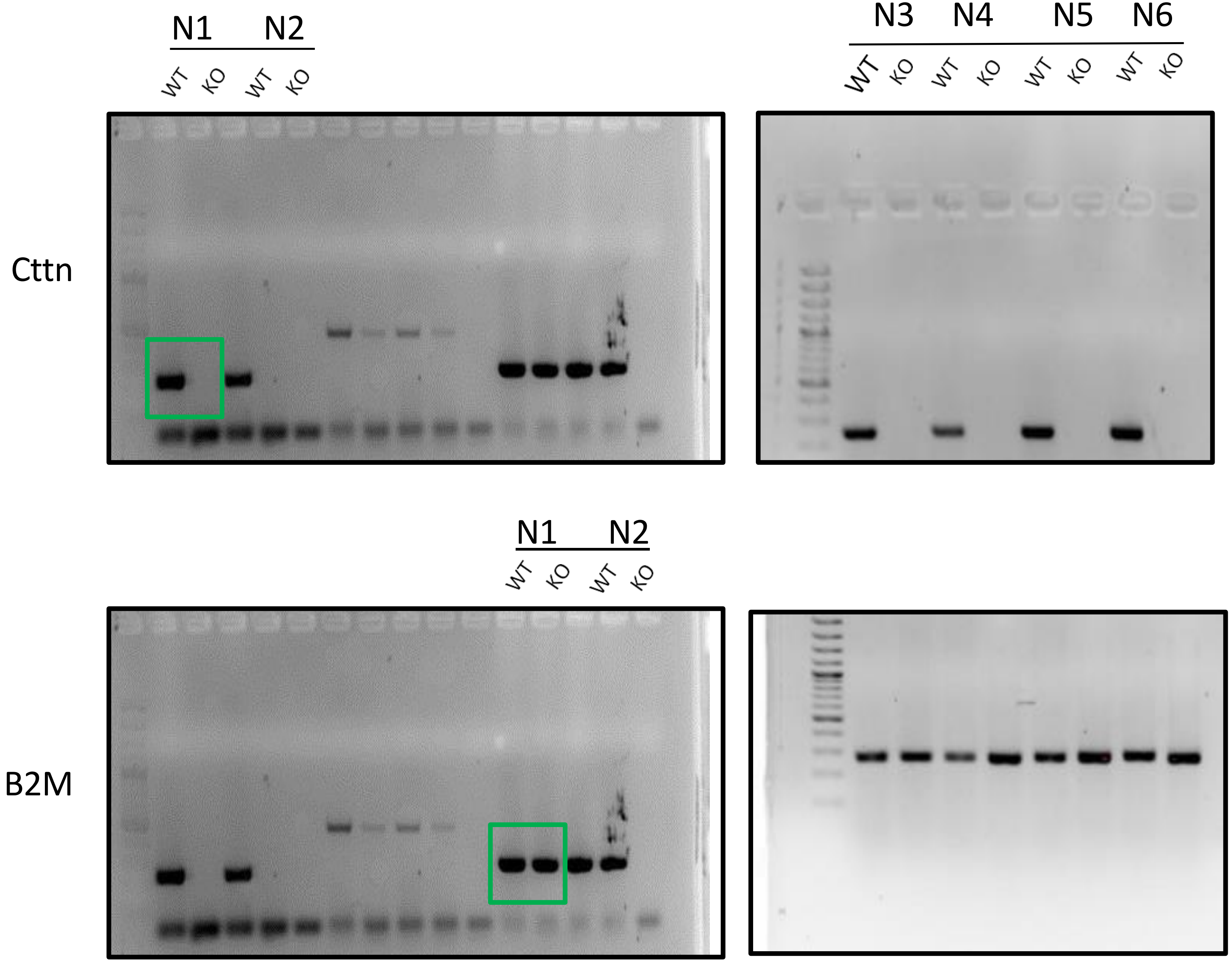
N1 N2
WT KO WT KO
N3 N4 N5 N6
WT KO WT KO WT KO WT KO
Cttn
N1 N2
WT KO WT KO
B2M

Figure S6, b)

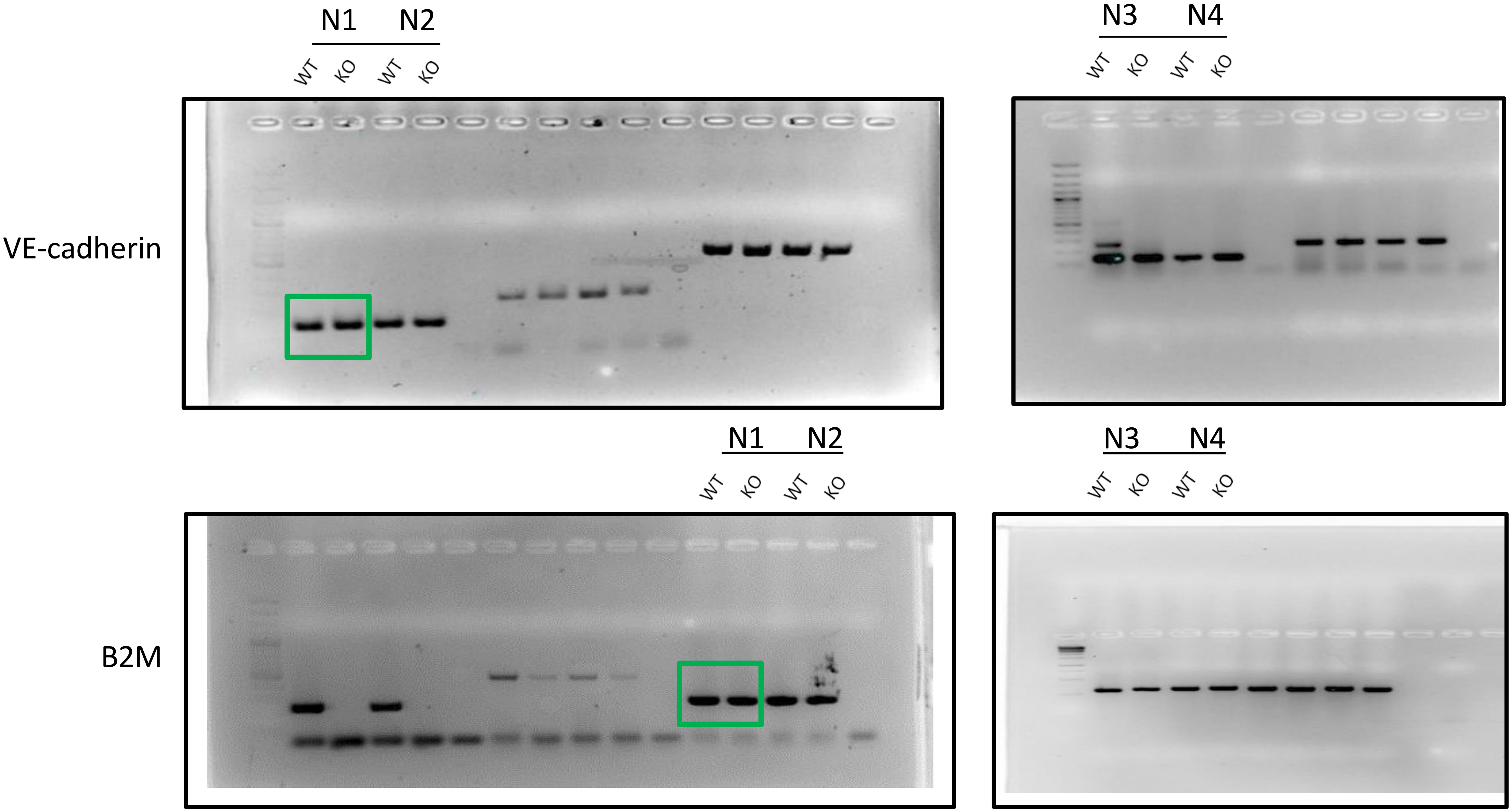
N1
N2
WT
KO
WT
KO
N3
N4
WT
KO
WT
KO
VE-cadherin
N1
N2
WT
KO
WT
KO
N3
N4
WT
KO
WT
KO
B2M

Figure S6, c)

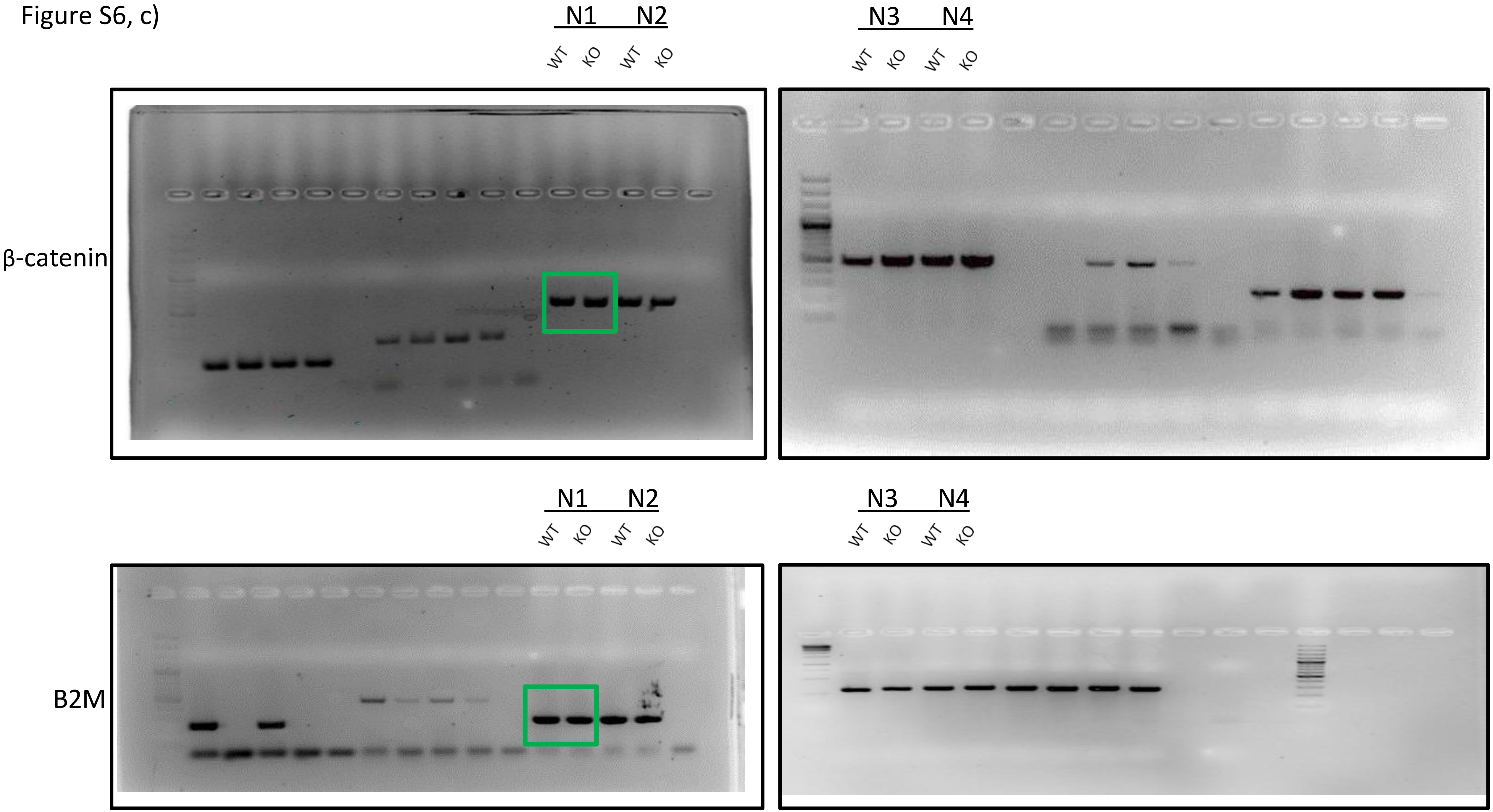
N1
N2
WT
KO
WT
KO
N3
N4
WT
KO
WT
KO
β-catenin
N1
N2
WT
KO
WT
KO
N3
N4
WT
KO
WT
KO
B2M

Figure S6, d)

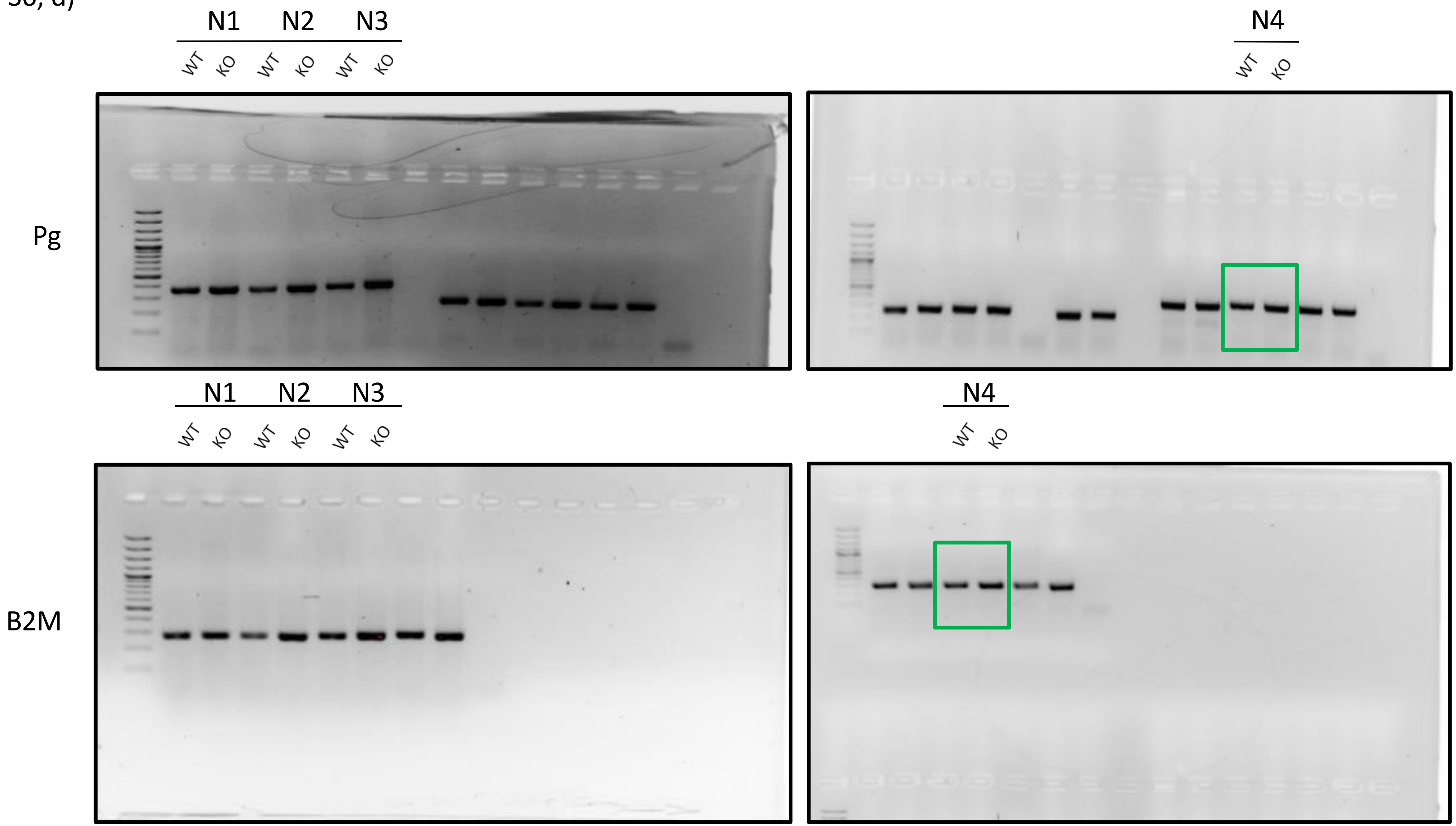
N1
N2
N3
WT
KO
WT
KO
WT
KO
N4
WT
KO
Pg
N1
N2
N3
WT
KO
WT
KO
WT
KO
N4
WT
KO
B2M

Figure S6, e)

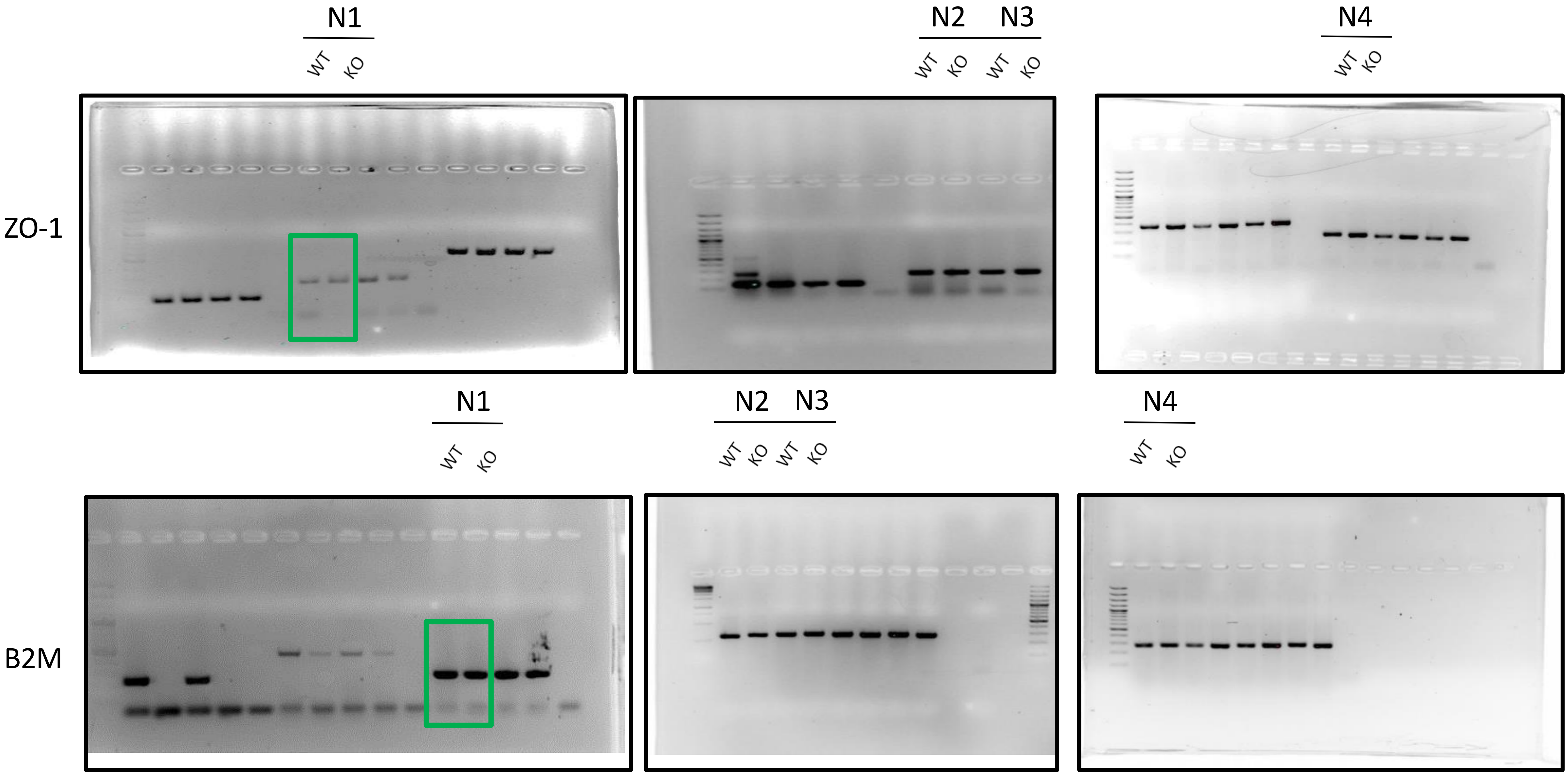
N1
WT KO
N2 N3
WT KO WT KO
N4
WT KO
ZO-1
N1
WT KO
N2 N3
WT KO WT KO
N4
WT KO
B2M

Figure S6, f)

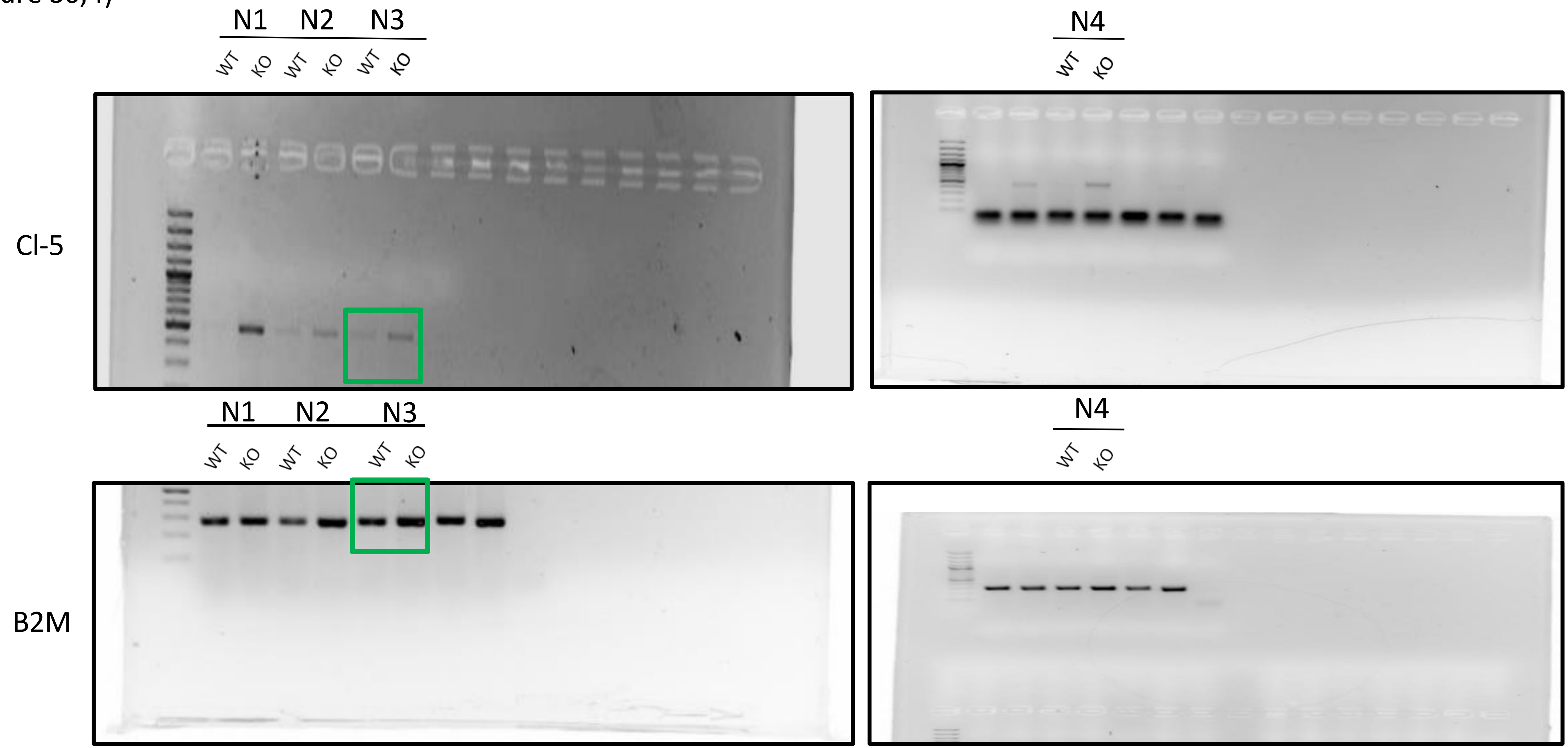

Figure S6, a-f) Original gel images for PCR. The green square determines the selected blot used in the figure 2.

Original Western blot gel images for VE-cadherin IPs

Figure S7, a)

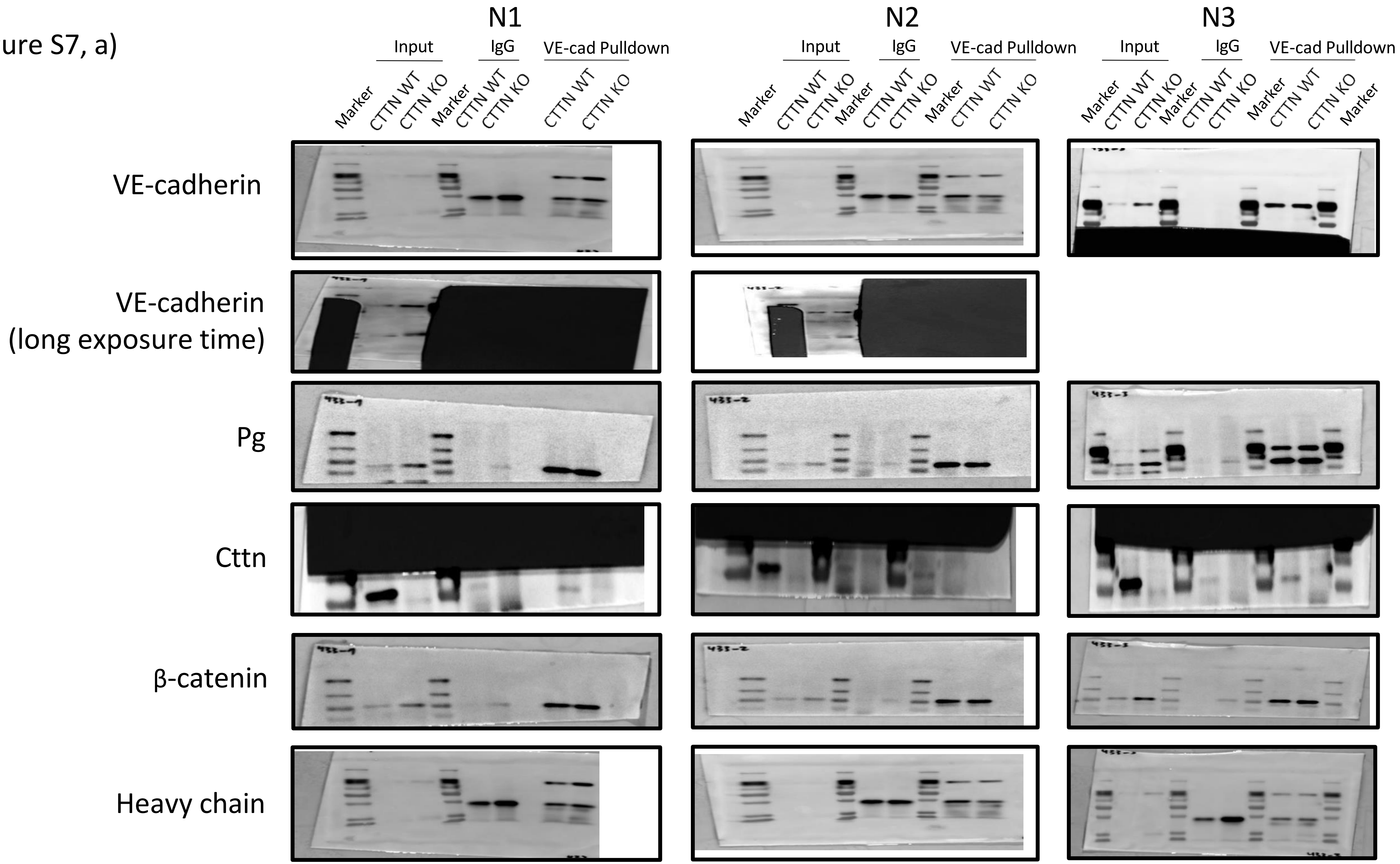
N1
N2
N3
Input
IgG
VE-cad Pulldown
Marker
CTTN WT
CTTN KO
VE-cadherin
VE-cadherin (long exposure time)
Pg
Cttn
β-catenin
Heavy chain

Figure S7, b)

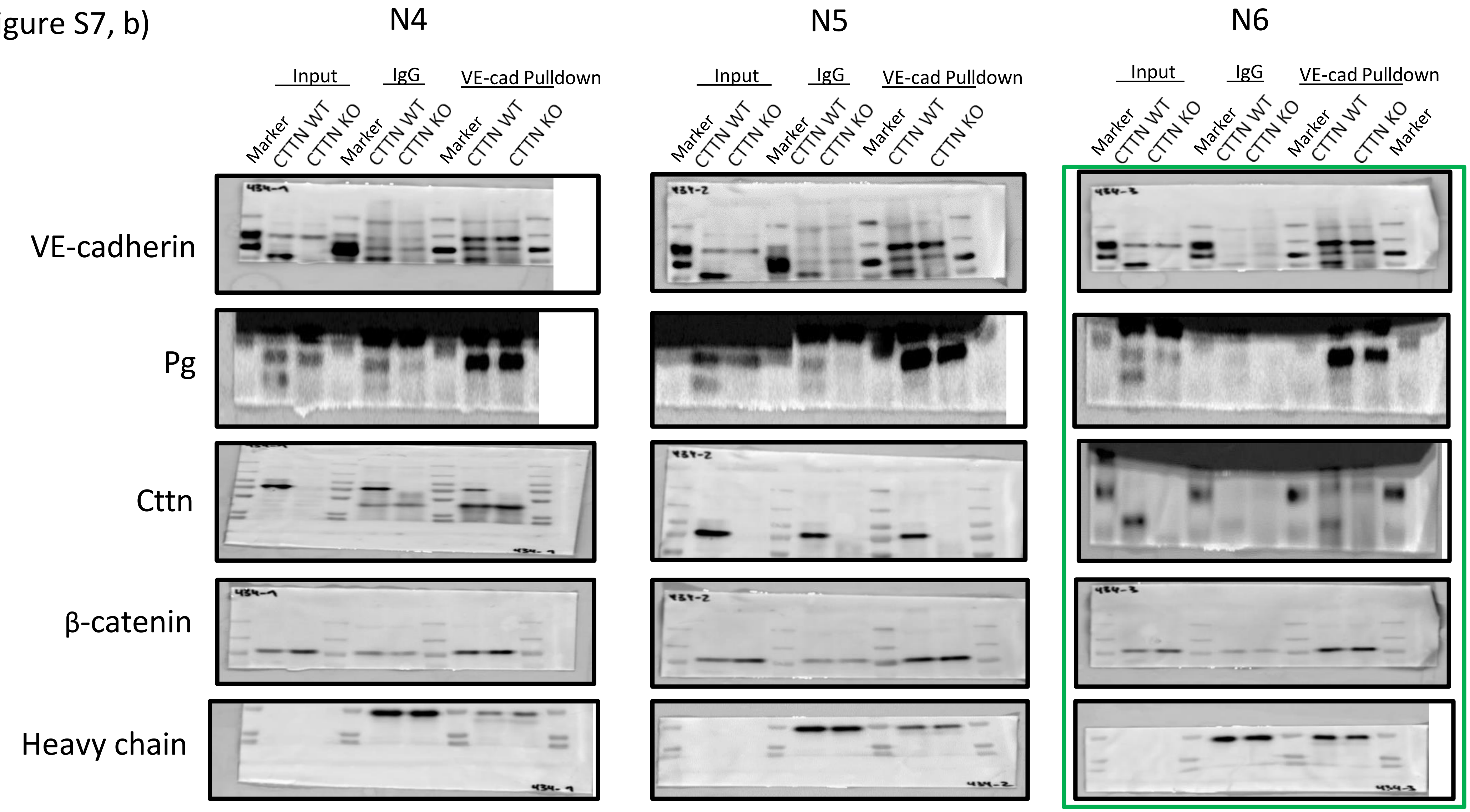

Figure S7, a-b) Original gel images for VE-cadherin IPs. To prevent over exposure, the black film was used to cover the bands. The green square determines the selected blots used in the figure 3.

Original WB gel images for Vehicle or F/R treatment

Figure S8, a)

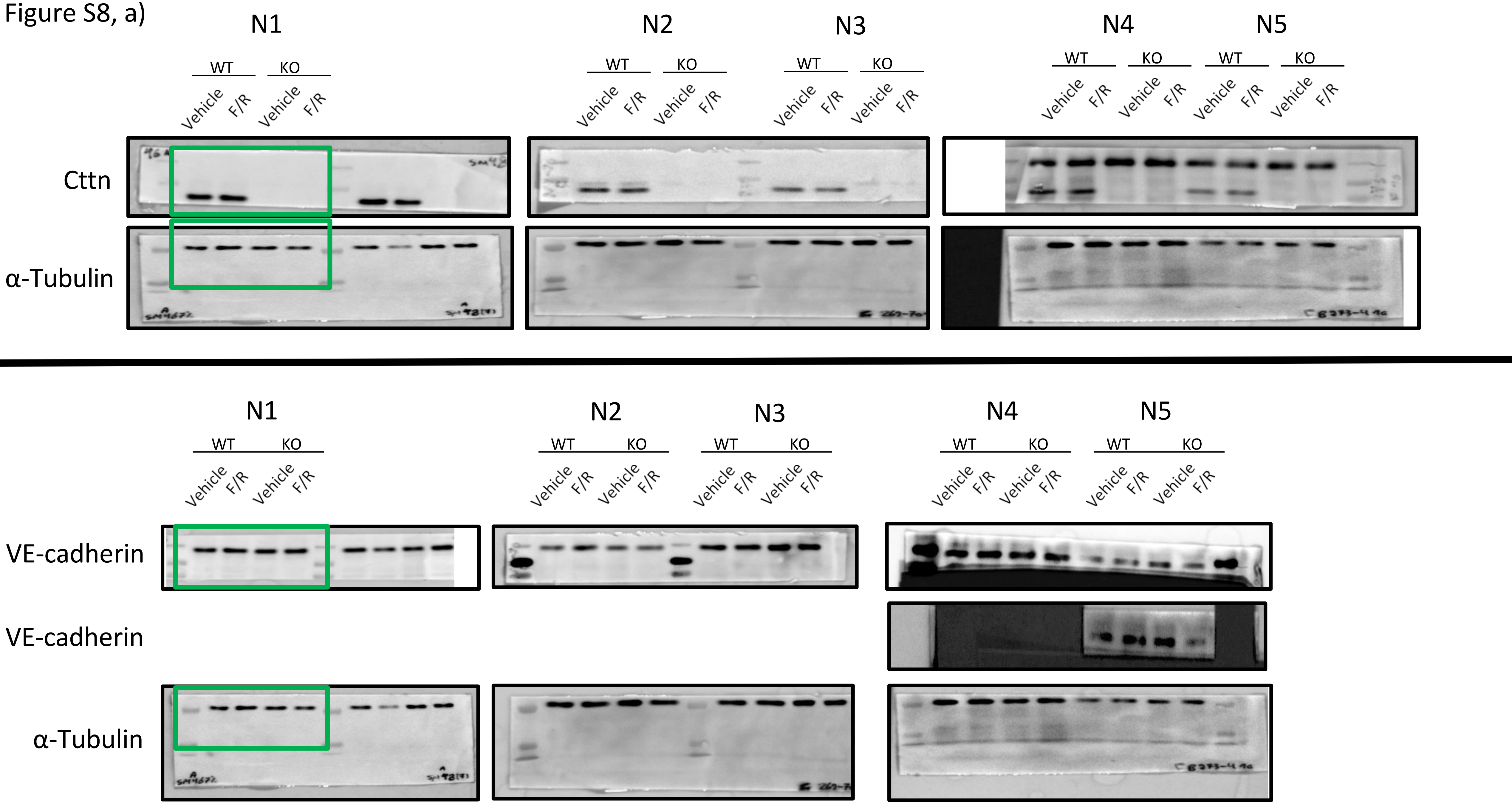
N1
N2
N3
N4
N5
WT
KO
Vehicle
F/R
Cttn
α-Tubulin
VE-cadherin
VE-cadherin
α-Tubulin

Figure S8, b)

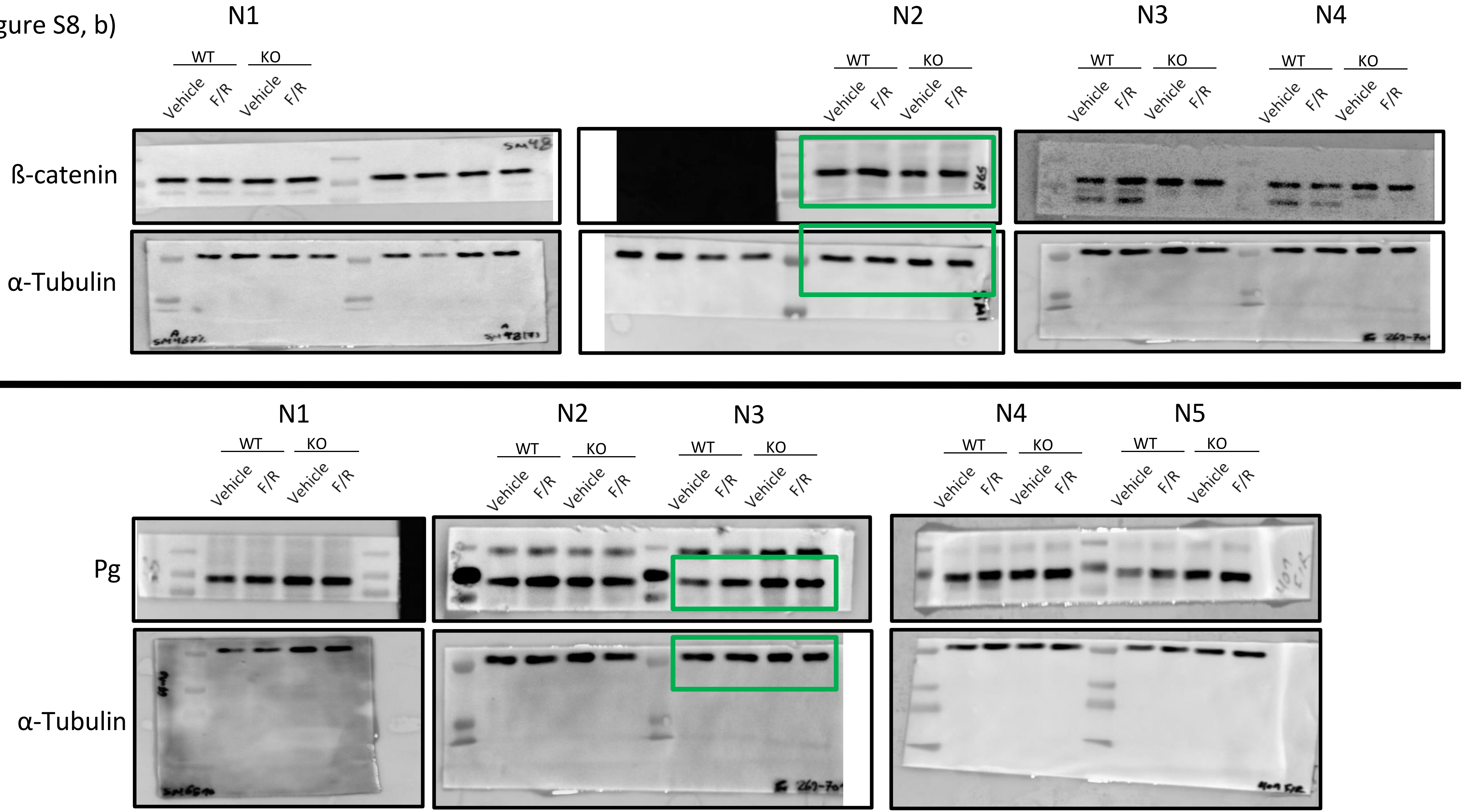

Figure S8, c)

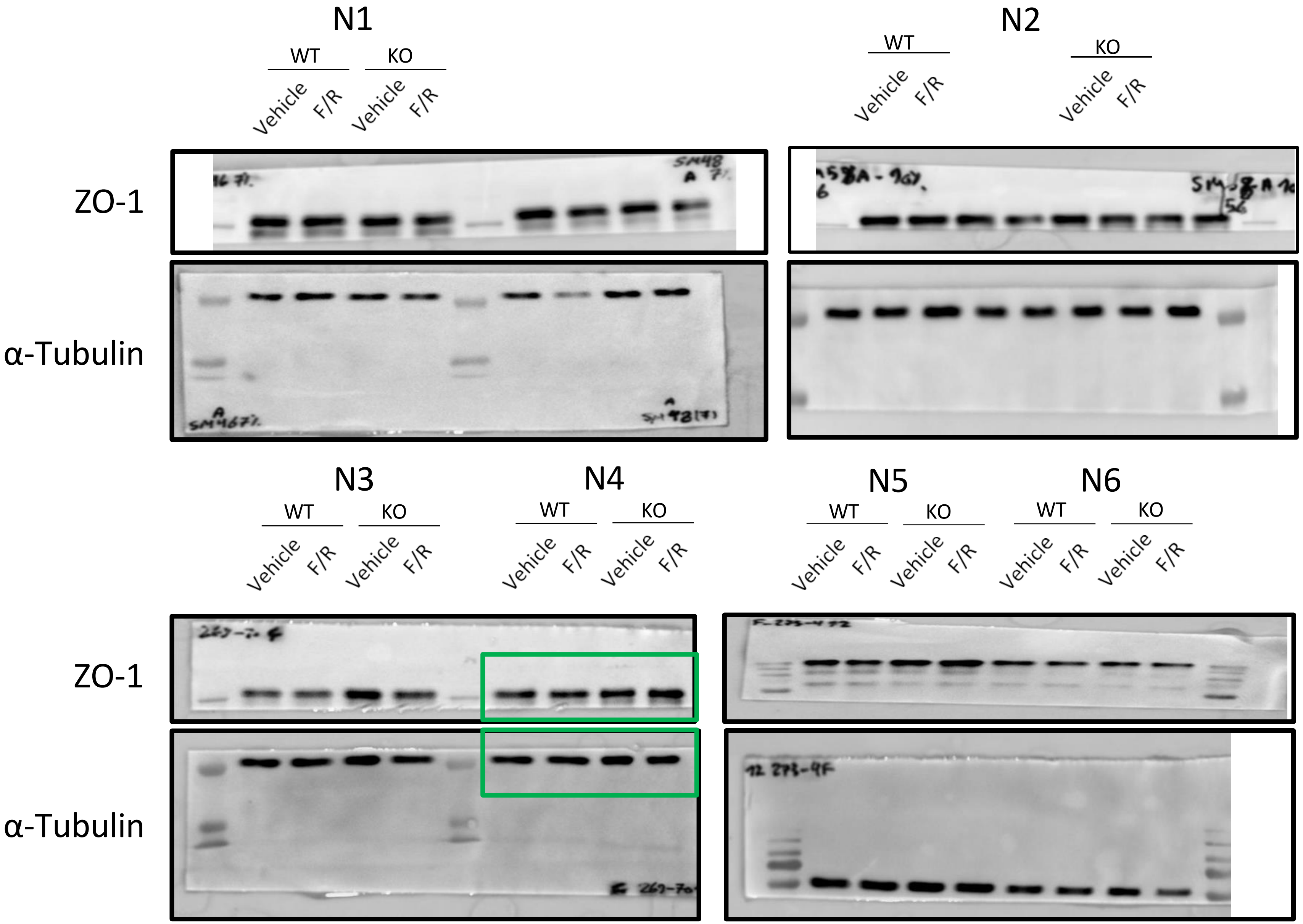

Figure S8, a-c) Original Western blot gel images for vehicle or F/R treatment. To prevent over exposure, the black film was used to cover the bands. The green square determines the selected blot used in the figure 4.

Figure S9, a)

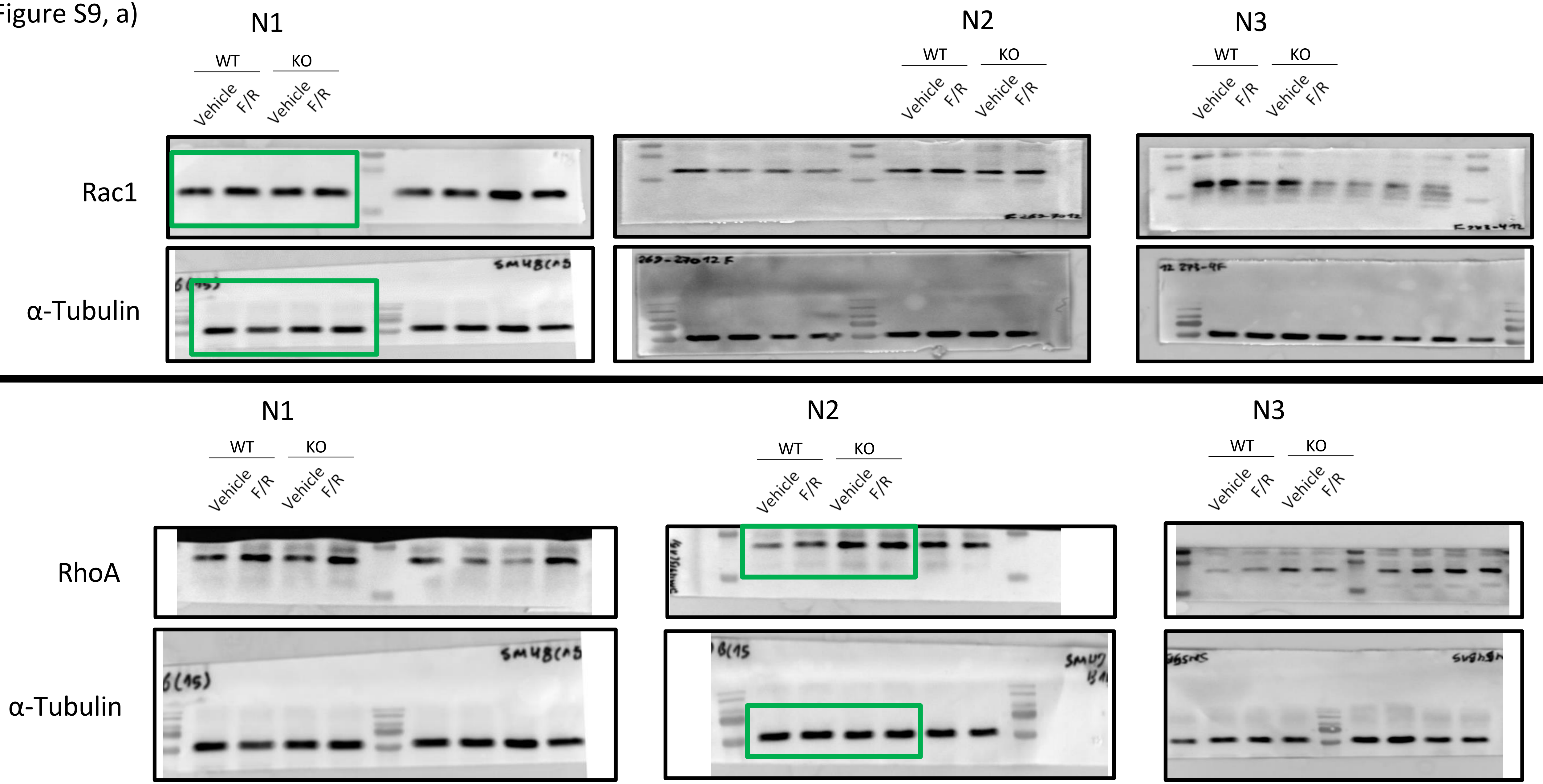

N1
WT
KO
Vehicle
F/R
Vehicle
F/R
N2
WT
KO
Vehicle
F/R
Vehicle
F/R
N3
WT
KO
Vehicle
F/R
Vehicle
F/R
Rac1
α-Tubulin
N1
WT
KO
Vehicle
F/R
Vehicle
F/R
N2
WT
KO
Vehicle
F/R
Vehicle
F/R
N3
WT
KO
Vehicle
F/R
Vehicle
F/R
RhoA
α-Tubulin

Original Western blot gel images for Rap1 pulldown

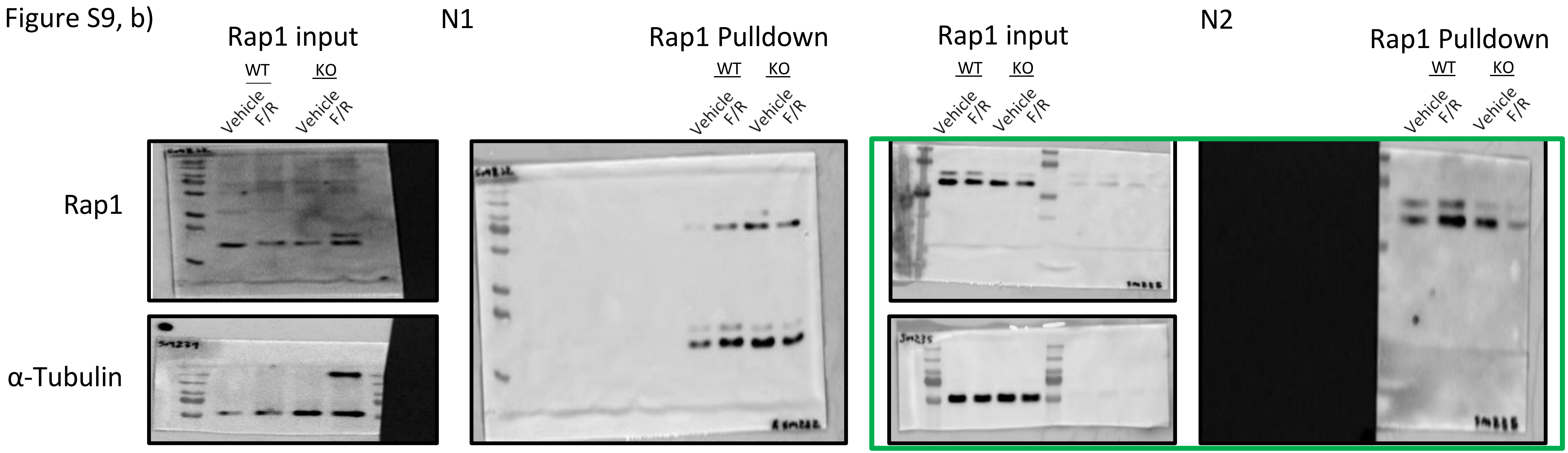

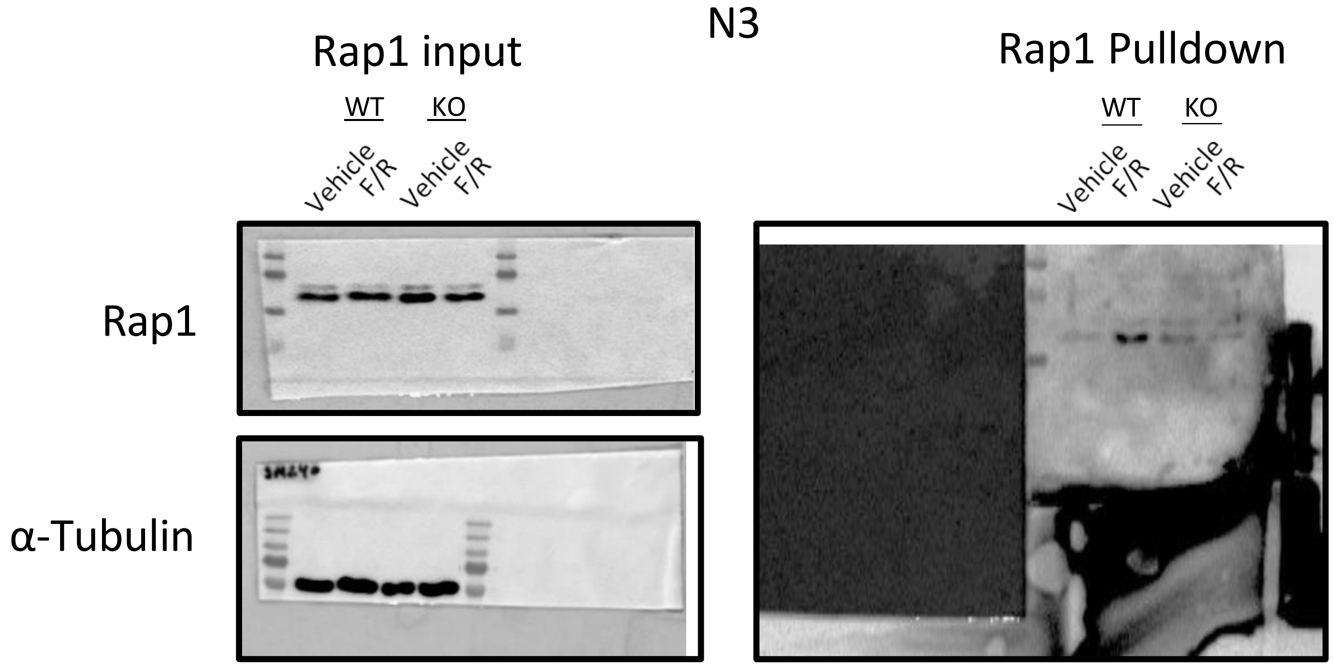

Figure S9, a-b) Original Western blot gel images for Rac1, RhoA and for Rap1 pulldown after vehicle or F/R treatment. To prevent over exposure, the black film was used to cover the bands. The green square determines the selected blot used in the figure 6.

Original Western blot images for Vehicle or CN04 treatment

Figure S10,a)

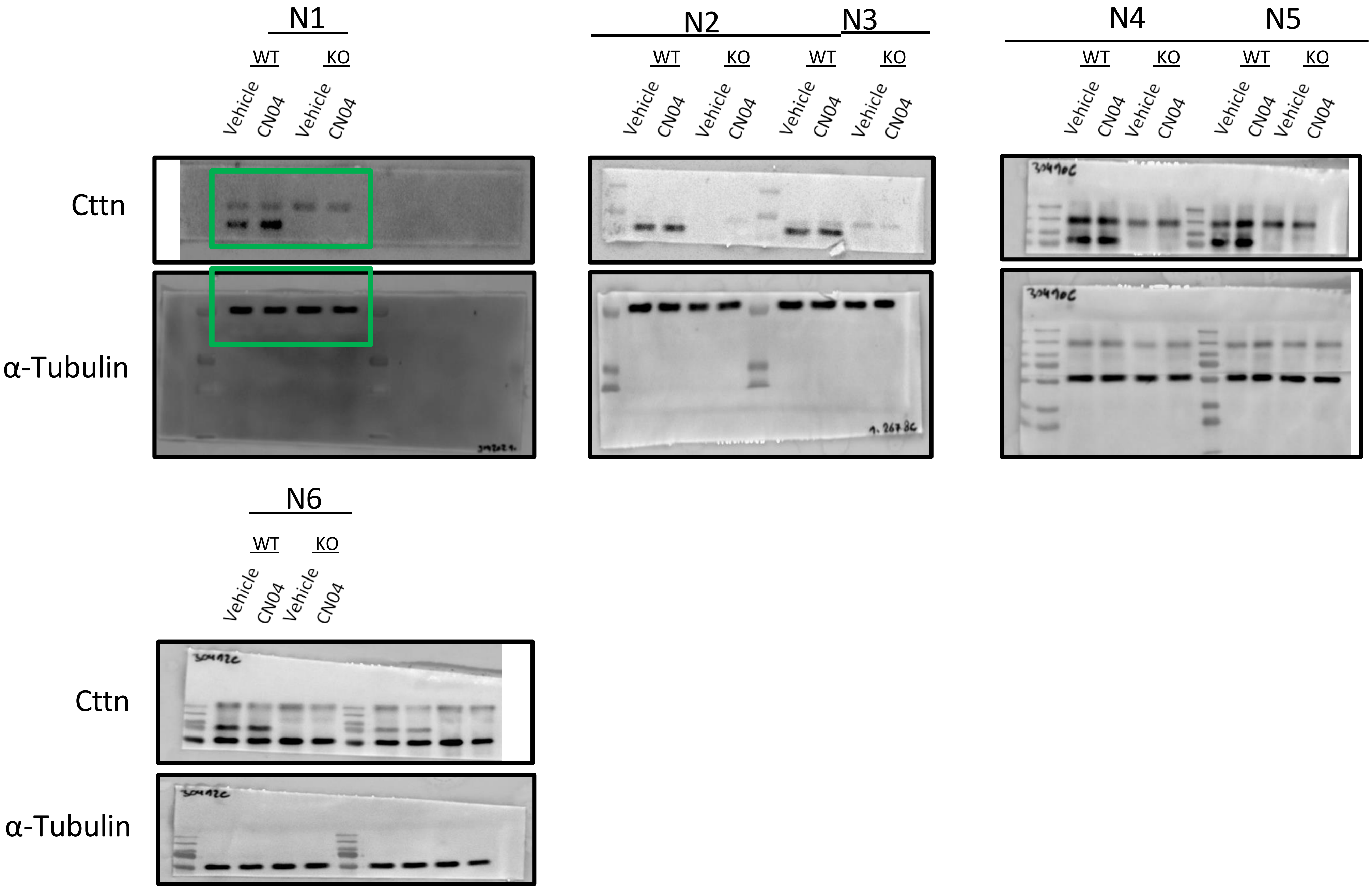

N1
WT
KO
Vehicle
CN04
Vehicle
CN04
N2
N3
WT
KO
WT
KO
Vehicle
CN04
Vehicle
CN04
Vehicle
CN04
Vehicle
CN04
N4
N5
WT
KO
WT
KO
Vehicle
CN04
Vehicle
CN04
Vehicle
CN04
Vehicle
CN04
Cttn
α-Tubulin
N6
WT
KO
Vehicle
CN04
Vehicle
CN04
Cttn
α-Tubulin

Figure S10, b)

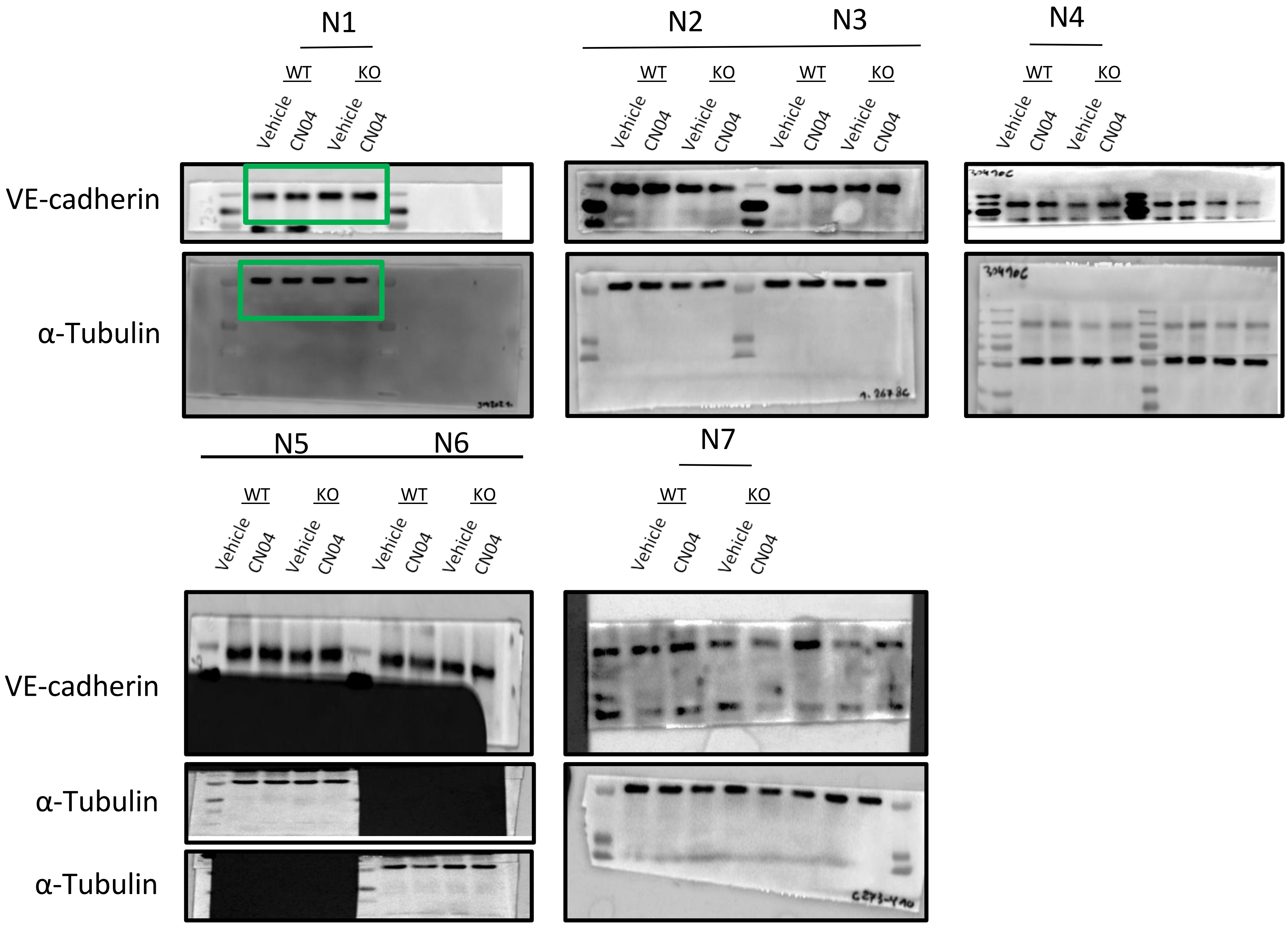

Figure S10, c)

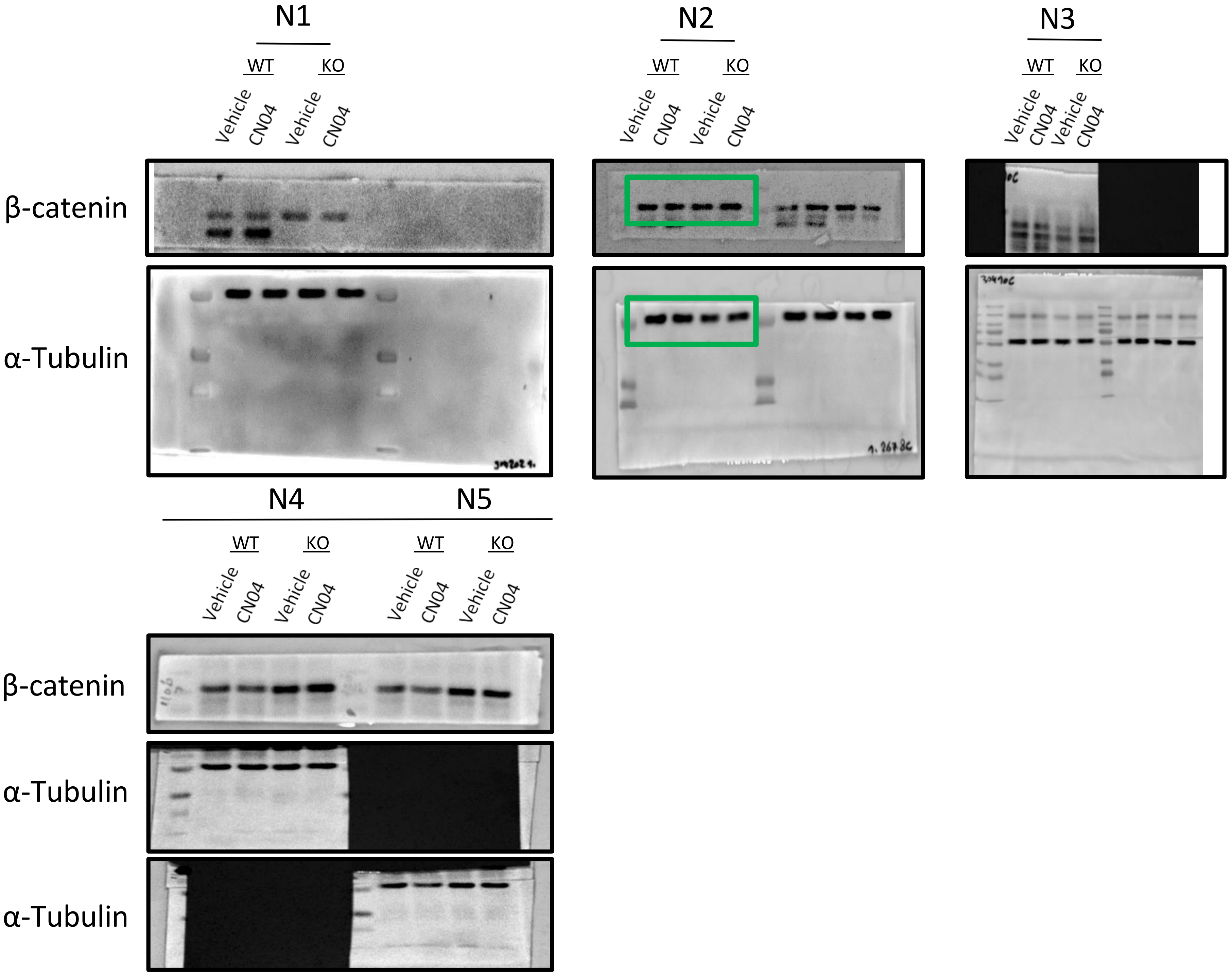

N1
WT
KO
Vehicle
CN04
Vehicle
CN04
N2
WT
KO
Vehicle
CN04
Vehicle
CN04
N3
WT
KO
Vehicle
CN04
Vehicle
CN04
β-catenin
α-Tubulin
N4
N5
WT
KO
WT
KO
Vehicle
CN04
Vehicle
CN04
Vehicle
CN04
Vehicle
CN04
β-catenin
α-Tubulin
α-Tubulin

Figure S10, d)

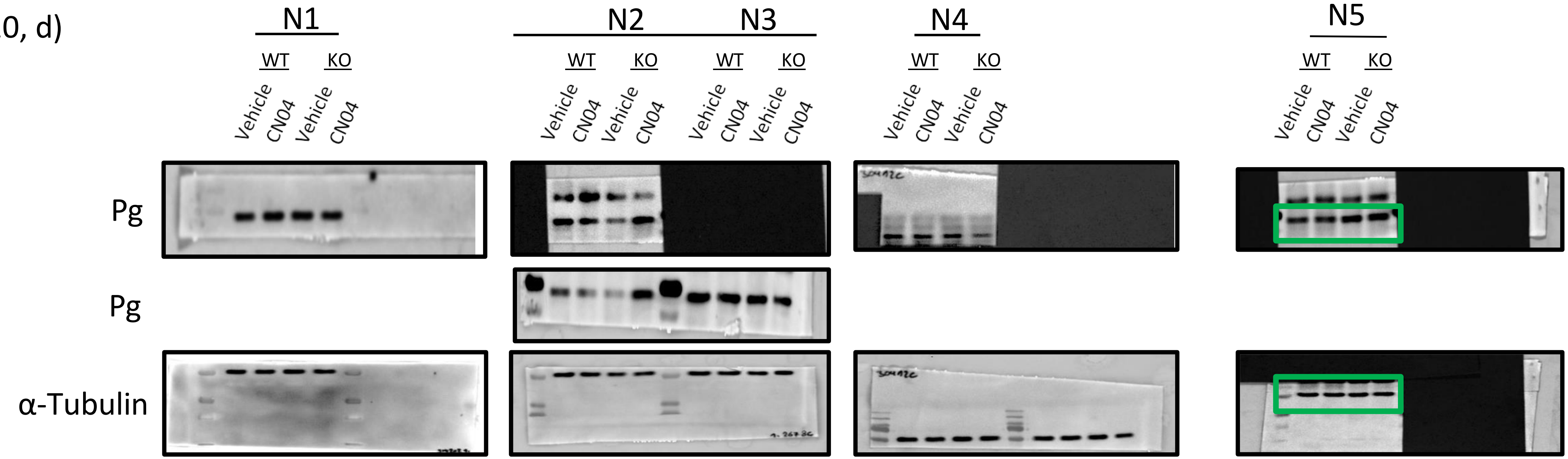

Figure S10, e)

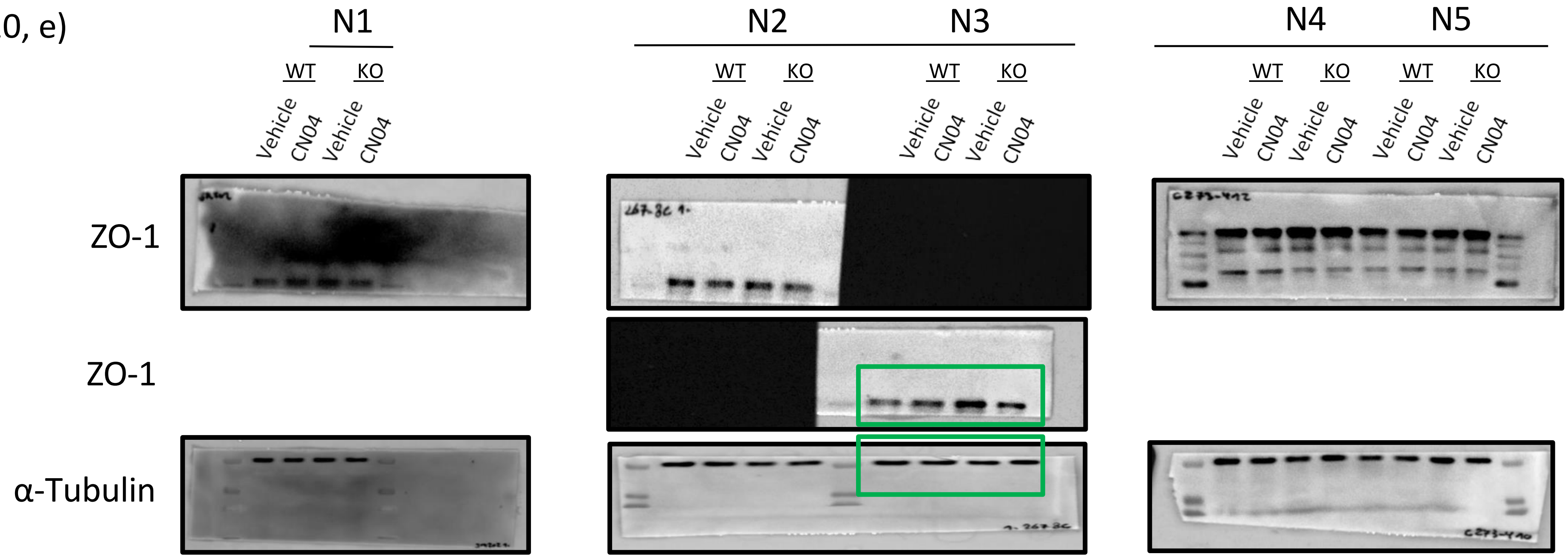

Figure S10, f)

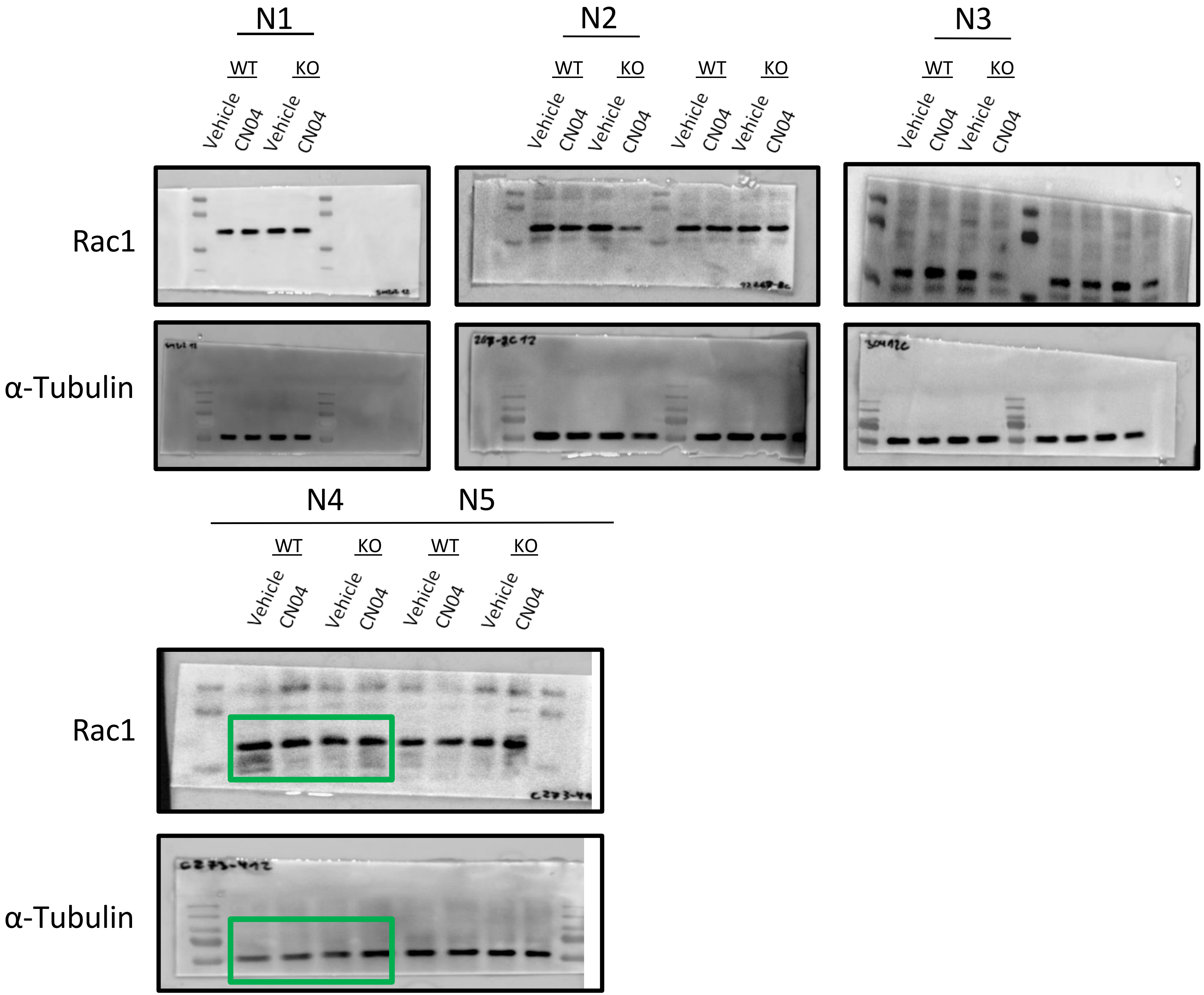

Figure S10, g)

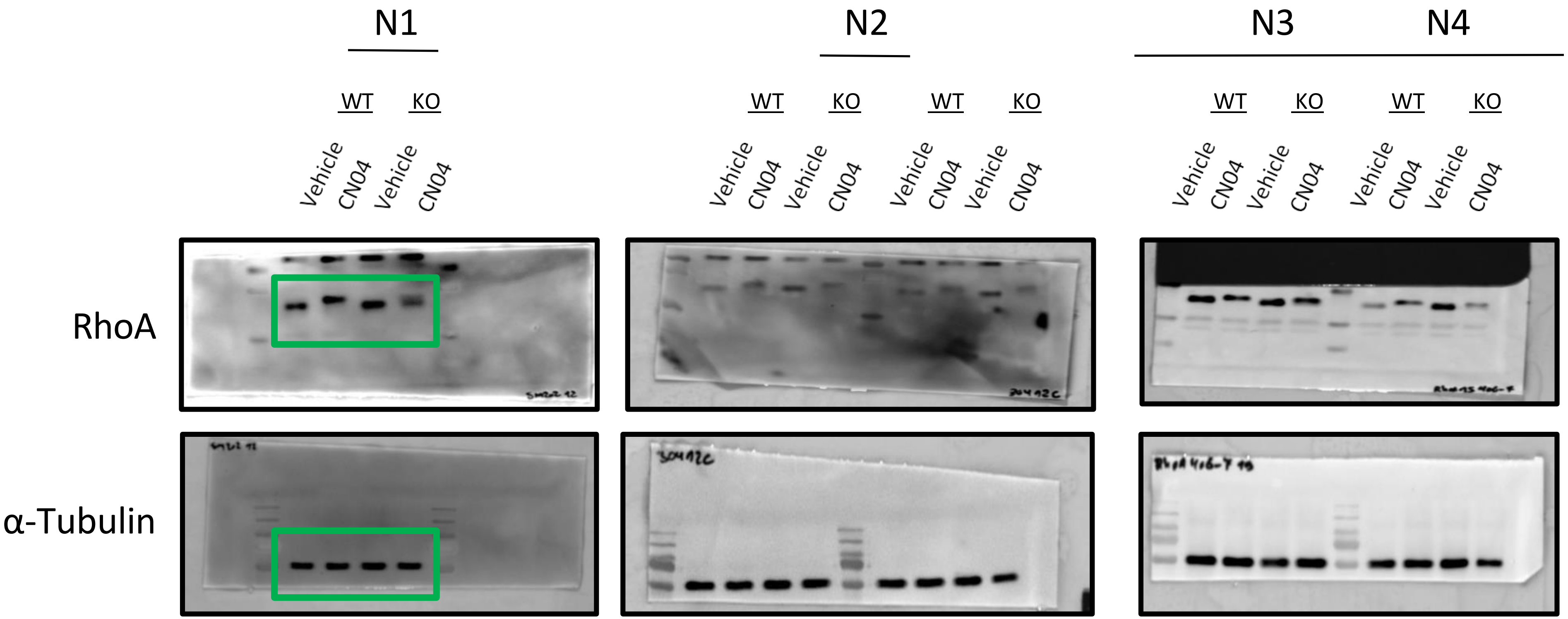

Figure S10, a-g) Original Western blot gel images for vehicle or CN04 treatment. To prevent over exposure, the black film was used to cover the bands. The green square determines the selected blot used in the figure 7.
